# Mining Natural Products with Anticancer Biological Activity through a Systems Biology Approach

**DOI:** 10.1155/2021/9993518

**Published:** 2021-08-12

**Authors:** Dionysia Theofylaktou, Işıl Takan, Gökhan Karakülah, Gökay Mehmet Biz, Vaso Zanni, Athanasia Pavlopoulou, Alexandros G. Georgakilas

**Affiliations:** ^1^DNA Damage Laboratory, Physics Department, School of Applied Mathematical and Physical Sciences, Zografou Campus, National Technical University of Athens (NTUA), 15780 Athens, Greece; ^2^Izmir Biomedicine and Genome Center (IBG), 35340 Balcova, Izmir, Turkey; ^3^Izmir International Biomedicine and Genome Institute, Dokuz Eylül University, 35340 Balcova, Izmir, Turkey; ^4^Department of Technical Programs, Izmir Vocational School, Dokuz Eylül University, Buca, Izmir, Turkey

## Abstract

Natural products, like turmeric, are considered powerful antioxidants which exhibit tumor-inhibiting activity and chemoradioprotective properties. Nowadays, there is a great demand for developing novel, affordable, efficacious, and effective anticancer drugs from natural resources. In the present study, we have employed a stringent in silico methodology to mine and finally propose a number of natural products, retrieved from the biomedical literature. Our main target was the systematic search of anticancer products as anticancer agents compatible to the human organism for future use. In this case and due to the great plethora of such products, we have followed stringent bioinformatics methodologies. Our results taken together suggest that natural products of a great diverse may exert cytotoxic effects in a maximum of the studied cancer cell lines. These natural compounds and active ingredients could possibly be combined to exert potential chemopreventive effects. Furthermore, in order to substantiate our findings and their application potency at a systems biology level, we have developed a representative, user-friendly, publicly accessible biodatabase, NaturaProDB, containing the retrieved natural resources, their active ingredients/fractional mixtures, the types of cancers that they affect, and the corresponding experimentally verified target genes.

## 1. Introduction

Cancer represents one of the leading causes of death globally. Despite the availability of anticancer therapeutics, cancer incidence is increasing gradually. According to the World Cancer Report of the International Agency for Research on Cancer (IARC) and the World Health Organization (WHO), the global cancer burden is estimated to have risen to 18.1 million new cases and 9.6 million deaths in 2018 (GLOBOCAN 2018 database: https://gco.iarc.fr/). Nowadays, research efforts are directed towards the discovery of naturally derived chemical compounds with anticancer potential [1]. A great number of highly potent bioactive compounds, derived from plants, have been found to possess anticancer properties, and this number is increasing exponentially [2, 3]. For example, there are natural products with antioxidant and anti-inflammatory capacities [4, 5], thereby preventing oxidative stress and inflammation, which can cause damage to DNA, eventually leading to genomic instability and eventually to carcinogenesis [6, 7].)

Natural products derived from plants and natural product-based anticancer drugs have been associated with reduced cancer mortality and risk. Fruits and vegetables contain vitamins, minerals, folate, plant sterols, carotenoids, and various phytochemicals such as flavonoid and polyphenols, which are suggested to have cancer chemopreventive potential [8–11].

Another category of plants that possess antitumor, antioxidant, and anti-inflammatory properties is spices and herbs [12]. They contain tannins, alkaloids, phenolic diterpenes, vitamins, flavonoids, and polyphenols. Spices and herbs such as curcumin, clove, rosemary, sage, oregano, and cinnamon are excellent sources of antioxidants due to the high content of phenolic compounds [8, 13, 14]. The antioxidants in edible and medicinal plant extracts have been shown to counteract ROS-mediated damage in diverse human cancers [15].

Furthermore, nuts are enriched in nutrients of high biological value. Nuts contain high amounts of vegetable protein [16, 17] and fat, mostly unsaturated fatty acids [17, 18]. They are also rich in a variety of other nutrients, as well as dietary fiber [19], vitamins (e.g., folic acid, niacin, tocopherols, and vitamin B6), minerals (e.g., calcium, magnesium, and potassium), and many other bioactive constituents such as phytosterols [17, 20] and phenolic compounds [17, 21]. Among the dietary plants, nuts, like peanuts and walnuts, contain the highest total content of antioxidants [21]. Nuts are associated with cancer prevention and they exert their potential chemopreventive effect through documented anti-antioxidant, anti-inflammatory, proapoptotic, antiproliferation, and antimetastatic activities [22].

Notably, natural compounds with cancer chemopreventive potential have also been derived from nonplant resources. For example, many cyclic peptides and analogues derived from marine organisms have been shown to possess anticancer, antimicrobial, anti-inflammatory, antiproliferative, and antihypertensive properties [23–25]. In addition, lactoferrin, a multifunctional protein found in bovine and camel milk, possesses anticancer, antimicrobial, and immune system boosting effects [26–29].

In the present work, taking into account the numerous studies on the biological (chemopreventive) activities of natural products, we have employed an in silico methodology to determine an effective combination of such products, retrieved from the biomedical literature, affecting diverse types of cancers and cancer-related pathways. Furthermore, we have developed a representative database, NaturaProDB, containing the retrieved data, which is maintained by the National Technical University of Athens, Greece.

## 2. Materials and Methods

### 2.1. Data Acquisition and Compilation

To obtain a broad spectrum of naturally occurring products with demonstrated anticancer activity, the bibliographic database MEDLINE/PubMed (https://www.ncbi.nlm.nih.gov/pubmed) was searched manually for full-text articles (from January 2003 up to 20 October 2020) using relevant keywords, including “natural products” or “naturally occurring compounds” or “natural ingredients” or “natural agents” or “natural substances” or “natural extracts” or “superfoods” and “cancer” or “malignancy.” The criteria applied to assess the anticancer potential of the natural products/fractional extracts were based on their ability to (i) suppress the growth of cancer cell lines and (ii) alter the expression of target genes, either oncogenes or tumor suppressor genes. Genus and species names (binomial nomenclature) were assigned to the source organism of the natural products according to the NCBI Taxonomy database [30]. The different types of cancers were classified according to NCBI's MeSH [31]. The official HUGO Gene Nomenclature Committee (HGNC) [32] symbols were used for the human genes.

The adverse effects of the natural compounds/extracts on human health were also assessed via extensive literature mining using the keywords “adverse effect” or “toxic∗” or “side effect” and “natural product.” The articles retrieved from MEDLINE/PubMed were carefully examined for any association between the active compounds/extracts and toxicity in humans.

### 2.2. Pathway Enrichment Analysis

The retrieved target genes were provided as input to WebGestalt (WEB-based GEne SeT AnaLysis Toolkit) [33], an online tool for functional annotation enrichment analysis, to identify statistically significant overrepresented cancer-associated WikiPathways. The threshold for the FDR-adjusted *p* value was set at 10^−3^, and hypergeometric distribution was used.

### 2.3. Functional Association Network

The associations among the genes/proteins under study were investigated and visualized with the usage of STRING (Search Tool for Retrieval of Interacting Genes/Proteins) v11.0 [34, 35], a database of either known or predicted, direct or indirect, association data among genes or proteins. These data are derived from diverse resources, including text mining of the scientific literature, biological and biochemical pathways, gene coexpression, high-throughput experiments, and gene fusion. The confidence score for displaying interactions was set to 0.9.

The software platform Cytoscape (http://www.cytoscape.org/) [36] was used for network analysis, processing, and visualization.

### 2.4. Statistical Analyses

All statistical analyses were performed with the R package “Stats” and Microsoft Excel Macros.

To identify the minimum number of superfoods that target all cancer types, as well as the maximum number of target genes, we utilized the R package “RcppGreedySetCover” for resolving set cover problems.

### 2.5. Differential Gene Expression Analysis

RNA sequencing (RNA-seq) gene expression data for 27 tumor and corresponding normal tissue samples (Table S1), from the TCGA and GTEx databases, respectively, were downloaded from the GEPIA2 (Gene Expression Profiling Interactive Analysis) online web server [37] (http://gepia2.cancer-pku.cn/). The differentially expressed genes (DEGs) between tumor and normal samples were identified using one-way analysis of variance (ANOVA), by setting the cutoff value for absolute log fold change ∣log_2_FC | ≥2 and FDR-adjusted *p* value ≤ 0.05.

### 2.6. Database Design

#### 2.6.1. Database Storage

“Cloud Firestore,” a NoSQL cloud database, was used to store and sync data for the client- and server-side web development. Cloud Firestore real-time data read-write feature was used for automatic data synchronization, thereby providing the user with the most updated data (https://firebase.google.com/docs/database).

#### 2.6.2. Website Design

Google's “Firebase Hosting” was utilized to host NaturaProDB's static assets (HTML and JavaScript); the popular front-end framework VueJS was offered through officially maintained supporting packages for creating the website user interface. The generated networks were processed and analyzed by Cytoscape (http://www.cytoscape.org/); Cytoscape.js library (https://js.cytoscape.org/) was used for network implementation, visualization, and interaction in the designed user interface.

## 3. Results

### 3.1. Data Retrieval and Assembly

**Figure 1:**
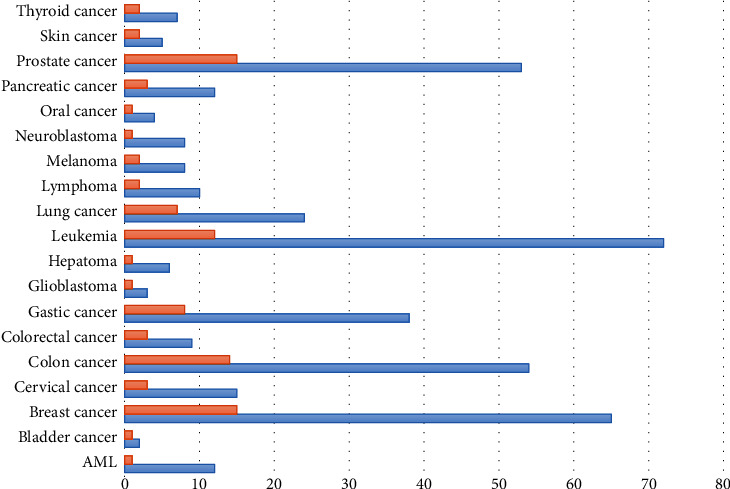
Bar graph depicting the distribution of natural products (orange) and genes (blue) with respect to cancer types.

A total of 562 relevant articles were selected after thorough review; of those, 86 articles were included in our study according to the eligibility criteria. Data collected from those studies regarding the scientific name of the source organism or food, natural product or fractional extract mixture, target cancer type, cell lines used for assessing anticancer activity in *in vitro* experimental studies, and target gene symbol along with its expression status (up- or down-regulated) were merged and recorded in a table (Table S1). The natural products were divided into eight major groups: vegetables/fruits, herbs, spices, nuts, dairy products, cereals, marine organisms, and oil. The vast majority of natural products originated from plants; however, natural compounds were also extracted from fungi, bacteria, marine organisms, and dairy products. Collectively, 87 source organisms/foods, 19 types of cancers, 105 target genes, and 66 cell lines were retrieved from the eligible studies and the relevant data were recorded in an Excel worksheet. The distribution pattern of natural products or target genes with respect to types of cancers is shown in Figure 1. The greatest percentages of natural products and target genes are distributed within the solid tumor breast and colon cancer, as well as the blood cancer leukemia.

**Figure 2:**
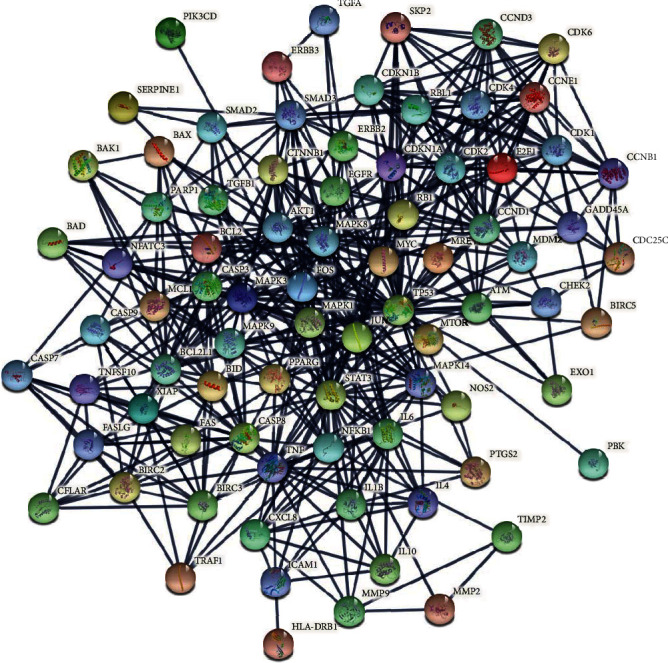
STRING output functional network of the 81 genes/gene products. The nodes represent molecules and the connecting lines (edges) indicate an interaction confidence score above 0.9.

**Figure 3:**
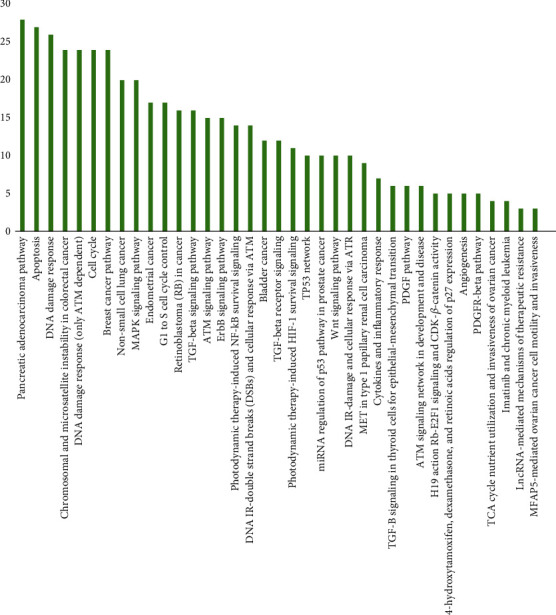
Bar plot depicting the overrepresented cancer-related pathways across target genes.

A total of 81 pivotal target genes were found to participate in cancer-relevant, interdependent pathways. These 81 genes and their corresponding proteins appear to be highly interconnected within the functional network shown in Figure 2. The generated network is quite dense, with an average node degree of 12.4 (Figure 2), suggesting tight associations, either physical or functional, among molecules so as to exert their antineoplastic effect. The distribution of genes across pathways is depicted in Figure 3. Among the overrepresented pathways are those associated with genomic instability, prosurvival pathways, TP53-mediated signaling, apoptosis, and cell cycle.

**Figure 4:**
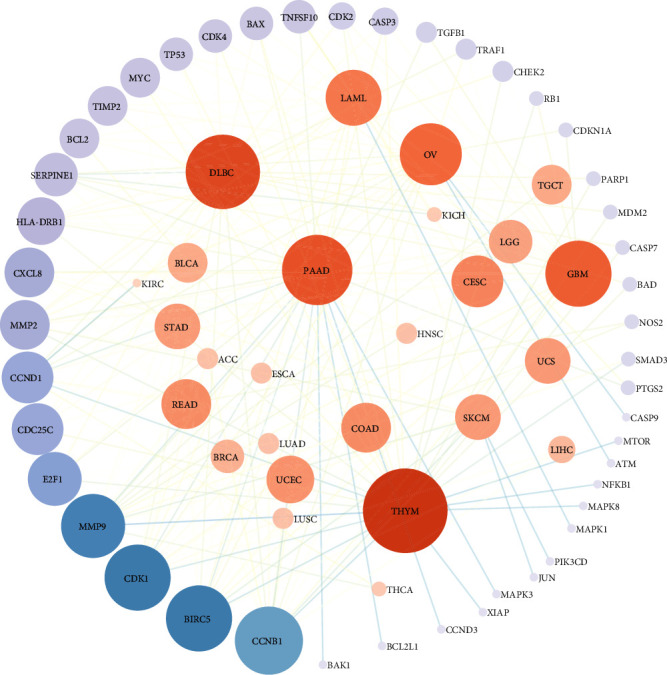
Bipartite network displaying TCGA-derived cancer-gene associations. The interactions are presented in a circular mode; the cancer types are shown at the center and the genes at the periphery. The size of the nodes is proportional to their connectivity degree.

We found a total of 22 natural foods targeting 57 genes (Table 1), most of them plants, except the fungus hazel mushroom. The active substances/functional extracts of the corresponding natural foods, as well as the effective concentration needed for cell growth inhibition, are listed in the second and third columns of Table 1, respectively. Based on literature research, no adverse health effects of the natural compounds/functional extracts were reported for the same dose range as in the one in the third columns in Table 1. In addition, a bipartite network was generated (Figure 4) based on the identified DEGs in diverse cancer tissue samples (Table 2). Collectively, 45 out of the 57 target genes appear to be highly connected due to their differential coexpression in 27 types of cancers (Figure 4).

Among the targeted genes are the key proapoptotic BAX (BCL2-associated X protein) [38], the caspases CASP3/8/9, and the cardinal player TP53 which are consistently upregulated in diverse cancer cell lines following treatment with natural compounds (Table 1). Furthermore, the antiapoptotic genes BCL2 and XIAP (X-linked inhibitor of apoptosis) [39, 40] are downregulated by natural products and upregulated in several cancer tissues (Tables 1 and 2). BCL2 can also suppress apoptosis by inhibiting the activity of caspases indispensable for apoptosis, such as CASP3 [41, 42]. The DNA damage response-associated gene PARP1 (poly(ADP-ribose) polymerase 1), pharmacological inhibitors of which are used in anticancer treatment [43], and CDKN1A (cyclin dependent kinase inhibitor 1A), a universal inhibitor of CDK/cyclin complexes [44, 45], are involved in DNA damage detection and DNA damage-induced cell cycle arrest [44, 46], respectively (Table 1). In addition, several natural products can potentially exert their antineoplastic effect on the oncogene *AKT1* (*AKT serine/threonine kinase 1*) [47]. Among the genes that are targeted by the 22 superfoods is *NFKB1*, which plays a protagonistic role in inflammatory responses [48, 49] (Table 1). NFKB1 also plays a dual role in apoptosis, either as inducer or as inhibitor of apoptosis [50]; thus, *NFKB1* was found to be both up- or downregulated by natural compounds in different cancers, as well as the same type of cancer (Table S1).

### 3.2. NaturaProDB

**Figure 5:**
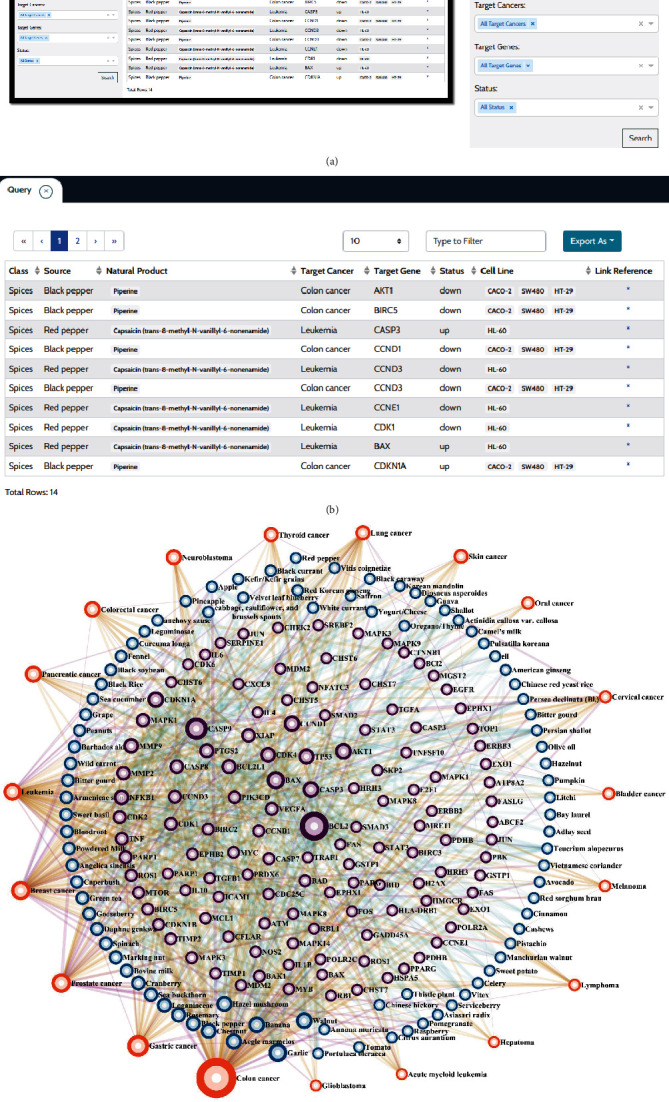
NaturaProDB workflow showing the (a) example input query, (b) example results page, and (c) “Natural Products Network,” where the node size is proportional to their connectivity degree.

The data presented in Table S1 were deposited in a repository, referred to as the National Technical University of Athens Anticancer Products Database (NaturaProDB; https://naturaprodb.web.app/). This database has a user friendly interface and can be queried using different options, that is, by (a) source, (b) natural product, (c) target cancer, (d) target gene, and (e) expression status of target gene, as well as the combination of the above options (Figure 5(a)). The results appear in a new window, in a tabulated format. Each entry contains the (i) general class of natural sources, (ii) food or organism, (iii) constituent compound/functional extract, (iv) target cancer, (v) target gene, (vi) gene status (up- or downregulated), (vii) cell line tested, and (viii) a hyperlink to the corresponding PubMed webpage (Figure 5(b)). The search output is provided in a JSON or TSV format. By clicking on the “Networks” tab, two interactive networks are displayed: the “TCGA Network” shown in Figure 4 and the “Natural Products Network” which includes food-target cancer-target gene associations (listed in Table S1). The latter network is highly interconnected, suggesting that natural products target multiple and diverse cancer cells and corresponding genes (Figure 5(c)).

## 4. Discussion

There is an ongoing need for alternative, effective, economical resources for drug development. Natural compounds with antineoplastic potential, as compared to synthetic compounds, are considered to be more efficacious and bioavailable and cost effective, with less toxic adverse effects [1]. The constant demand for natural antioncogenic medicines is also reflected in the number of databases dedicated to natural products with anticancer activity, such as CancerHSP [51], CHMIS-C [52], InPACdb [53], NPACT [54], and NCARE [55].

Another parameter that must be taken into consideration is the cellular, genetic, and metabolic heterogeneity of cancers and the complex tumor microenvironment. Designing of broad spectrum therapeutic strategies represents an intriguing solution to this problem. The so-called “dirty” drugs have multiple, instead of single, molecular targets. Within this context, the anticancer activity of several natural compounds could be safely combined in such a way as to maximize their inherent additive and synergistic effects and avoid any side effects, towards the design of potent dirty drugs. In our study, we suggest a combination of 22 natural foods which, based on in vitro experimental studies, could potentially target all 19 types of cancers and 57 key cancer-relevant genes under investigation. These genes are implicated in interconnected pathways (Figure 2), including those related to genomic integrity and cell cycle control. In particular, chromosomal instability and DNA damage response and repair (DDR/R) pathways are known to contribute largely to carcinogenesis. One of the characteristics of cancer is the presence of genomic lesions, caused either directly or indirectly, through the generation of DNA-damaging intermediates, like reactive oxygen species (ROS) and in general free radicals (e.g., hydrogen ion and hydroxide) [56, 57]. These genomic lesions, if not properly processed, could lead to genomic instability and eventually to carcinogenesis [58]. ATM (Table 1) plays a protagonistic role in the initial stage of DDR/R, that is, DNA damage detection and stress-response signaling [59, 60]. ATM signaling is activated by a wide variety of DNA lesions and DNA replication stress [61, 62]. Cyclin D1 (CCND1) (Table 1) was demonstrated to induce post-DNA damage cell cycle arrest and apoptosis in different types of cancers [63, 64].

Moreover, several cell prosurvival pathways (Figure 2) were found to be enriched in the gene set. For example, the transforming growth factor beta (TGFB), MAPK, ERBB, HIF-1, WNT, PDGF/PDGFRB, and NFKB signaling pathways are implicated in several aspects of cancer initiation, promotion, and progression [65–71], by mediating survival of cancer cells. The apoptotic pathway is also overrepresented. Cancer cells have developed the ability to evade apoptosis, mainly due to deregulation of key proapoptotic molecules like TP53, BAX, BCL2, BIRC5, and XIAP (Table 1).

Several of the plants (e.g., garlic, peanuts, spinach, and black soybean) listed in Table 1 contain more than one active compound or phytochemicals with anticancer activity (Table S1), suggesting that they exert their anticancer effect in an additive or synergistic manner. One of the garlic clove ingredients, the phenylamine NBNMA (Table 1), was shown to induce cell cycle arrest, via downregulation of the cell cycle M-phase inducer cyclin dependent kinase 2 (CDK2) and overexpression of CDKN1A, as well as apoptosis, by activation of the proapoptotic factors CASP3, -8, -9, BAX, and BAD and, conversely, inactivation of the antiapoptotic BCL2, BCL2L1, BIRC2, and XIAP in leukemic cells [72]. As it is shown in Table 2, BCL2 is overexpressed in acute myeloid leukemia. Another garlic compound, allicin was shown to sensitize hepatocellular carcinoma cells to anticancer agents via a ROS-dependent signaling pathway [73]. Allicin, also, increases the sensitivity of colorectal cancer cells to radiation through the inhibition of a NFKB1-mediated pathway [74]. Furthermore, allicin potentiates apoptosis in human glioblastoma cells, by elevating the expression of BAX and downregulating BCL2 (Table 1) [75]. Peanuts, which contain procyanidins in their skin, can inhibit the proliferation of prostate cancer cells and promote apoptotic cell death by downregulating BCL2 and upregulating the proapoptotic factors BAX, CASP3, and the TP53 [76]. The phenolic compounds, which comprise the largest group of phytochemicals, are known to exert their antineoplastic effect by contributing to cell proliferation, apoptosis, angiogenesis, metastasis, and inflammation under oxidative stress [77–81]. The phenolic antioxidant resveratrol in peanuts (Table 1) was shown to exhibit antiproliferative activity in cervical and breast cancer cells, by decreasing the expression of the DNA damage-induced prosurvival protein kinases MAPK3 and CDK4 and, conversely, elevating TP53 and CDKN1A [82].

Juglone, a phenolic compound in the Manchurian walnut, was shown to inhibit the proliferation of human leukemia cells and enhance apoptosis [83]. In particular, Juglone (Table 1) markedly inhibited the phosphorylation of PI3K/AKT/mTOR, a major antiapoptotic and prosurvival signaling pathway which is overactivated in multiple cancers [84], and induced the cleavage and activation of the proapoptotic procaspase-3. Moreover, juglanin was shown to inhibit the proliferation of breast cancer cell proliferation through the differential regulation of cell cycle-associated proteins (i.e., CDC25C, CDK1, CDKN1B, and CHEK2), the activation of the proapoptotic factors BAD, BAX, CASP3, -8 and -9, and, conversely, the suppression of the antiapoptotic protein BCL2 [85]. Of note, CDK1 (cyclin-dependent kinase 1), which is downregulated by the flavonoid compound juglanin (Table 1), was found to be overexpressed in invasive breast cancer (Table 2); Izadi et al. suggest that CDK1 is the best CDK target for breast cancer therapy [86].

The cancer-preventive properties of the cruciferous vegetables (belonging to the family *Brassicaceae*) have been acknowledged for a long time. The active ingredient indole-3-carbinol (I3C) has been documented to play a role in the prevention of several cancers [87]. For example, I3C was found to inhibit the growth of prostate cancer cells and induce G1 cell cycle arrest and apoptotic cell death [88]. Furthermore, it was demonstrated by Takada and colleagues [89] that I3C (Table 1) blocks the expression of NFKB1-regulated prometastatic, proproliferation, and antiapoptotic gene products (i.e., AKT1, BCL2, BIRC2, BIRC5, CCND1, MMP9, PTGS2, TNF, and XIAP) in myeloid and leukemia cells. Of those genes, *BCL2* and *PTGS2* were shown to be upregulated in acute myeloid leukemia (Table 2). In the same study, the gene encoding NOS2 (nitric oxide synthase 2), which catalyzes the production of the reactive free radical nitric oxide, was downregulated, leading to the suggestion that I3C might exert antioxidant activity.

Flavonoids present in citrus fruits have been documented to exhibit cancer-preventive potential by participating in cell cycle inhibition, suppression of metastasis, and angiogenesis, as well as anti-inflammatory signal transduction pathways [90]. In particular, flavonoids isolated from *Citrus aurantium* were shown to inhibit the growth of human gastric cancer cells, by suppressing the proteins CCNB1 (cyclin B1) and CDK1 (Table 1) which control cell cycle progression; the corresponding genes *CCNB1* and *CDK1* were found to be upregulated in stomach cancer (Table 2). In addition, activation of CASP3 together with the inactivation of PARP1, which is involved in DNA damage repair, potentiated apoptosis [91].

The spice *Curcuma longa*, commonly known as turmeric or “Indian saffron,” has been used in the folk medicine of India for thousands of years. Curcumin, the major active ingredient of turmeric, is a powerful antioxidant, with well-documented anti-inflammatory and anticancer potential [92]. Curcumin was shown to suppress growth of bladder and pancreatic cancer cell lines through the inhibition of NFKB1-regulated proinflammatory and proproliferative gene products PTGS2 [93] and CXCL8 [94] (Table 1); both CXCL8 and PTGS2 were also found to be downregulated in pancreatic cancer (Table 2). Moreover, curcumin was demonstrated to inhibit metastasis in human papillary thyroid carcinoma cells by downregulating components of the prometastatic signaling pathway TGFB1/SMAD2/SMAD3 [95] (Table 1).

Other phytocompounds, like sporamin in sweet potato [96–98], piperine in black pepper [99], sanguinarine in bloodroot [100–102], 3-deoxyanthocyanins in red sorghum bran [103], and aloin in *Aloe vera* [104], have been documented to exhibit anticancer effects in several cancers. Of importance, these agents possess antioxidant properties [105–108]. Aloin [104] and piperine [109] can inhibit proliferation of (colo)rectal cancer cells; the genes *MYC* and *BIRC5*, the expression of which is decreased by the two compounds (Table 1), are otherwise overexpressed in colon adenocarcinoma (Table 2). Similarly, the antiapoptotic *BCL2L1* gene, which was found downregulated in sporaminin-induced apoptotic pancreatic cancer cells [97] (Table 1), is overexpressed in pancreatic adenocarcinoma (Table 2). Furthermore, *β*-caryophyllene and *β*-caryophyllene oxide extracted from *Aegle marmelos* are suggested to possess anti-inflammatory potential and were demonstrated to induce apoptosis in cancer cells of diverse tissue origin, that is, lymphoma (i.e., haematological cancer) and neuroblastoma (i.e., nerve tissue neoplasm), through overexpression of the proapoptotic (*ATM*, *BAK1*, *BAX*, and *CASP8/9*) and underexpression of the antiapoptotic (*BCL2*, *MDM2 proto-oncogene*, and *PTGS2*) genes [110] (Table 1). The genes *BCL2* and *MDM2* are also upregulated in lymphoid neoplasm diffuse large B-cell lymphoma (Table 2).

## 5. Conclusions

Nowadays, there is a great need for drugs derived from nature that can be 100% compatible if possible to the human organism, with a potential applicability in the fight against human diseases. In this study, we have targeted human cancer. Based on systematic searches and use of current bioinformatics methodologies, we have designed a biodatabase exceeding the existing standards of natural product databases, i.e., at a systems biology level. We have found that diverse natural products, with no observed adverse health effects, can target dissimilar cancer types through the significant alteration of the expression of multiple, common genes, which are involved in shared interconnected cancer-relevant pathways. Therefore, the efficient and safe combination of bioactive compounds derived from natural resources can be potentially applied to exert cytotoxic effects on diverse types of cancer cells and regulate the expression of numerous target genes which play a central role in cancer pathways. Further studies in animal models should be directed towards the investigation of the chemopreventive actions and safety of those compounds. The aforementioned findings should be taken into consideration in the rational design of drugs with broad spectrum activity, such as “dirty” drugs. Lastly, NaturaProDB was developed to facilitate the retrieval of relevant information. In conclusion, in this study, we provide a novel and effective systems biology approach to investigate the potential value of the combined activity of natural products in cancer chemoprevention, which can be exploited in the development of anticancer multitargeted therapies.

## Figures and Tables

**Table 1:** List of the selected organisms/foods, active compounds/extracts, effective inhibitory concentration, affected cancer type, and target genes.

Source	Active substance/extract	Effective concentration	Cancer type	Target gene
*Actinidia callosa* var. callosa	Ethyl acetate fraction of *Actinidia callosa* var. callosa (EAAC)	IC_50_: 73.3 and 92.7 *μ*g/ml for 24 h treatment	Hepatoma	AKT1, MAPK1, MMP2, MMP9, PIK3CD, TIMP2
*Aegle marmelos*	Beta-caryophyllene and caryophyllene oxide fractions of *Aegle marmelos* extract	Maximum inhibition: 50 *μ*g/ml for 24 h treatment	Lymphoma; neuroblastoma	ATM, BAK1, BAX, BCL2, CASP8, CASP9, MDM2, PTGS2
*Allium sativum* (garlic)	Allicin	Maximum inhibition: 90 *μ*g/ml for 24 h treatment	Glioblastoma	BAX, BCL2
*Allium sativum* (garlic)	N-Benzyl-N-methyldecan-1-amine (NBNMA)	Maximum inhibition: 50 *μ*g/ml for 24 h treatment	Leukemia	BAD, BAX, BCL2, BCL2L1, BIRC2, CASP3, CASP8, CASP9, CDK2, CDKN1A, XIAP
*Aloe vera* (Barbados aloe)	Aloin (AL)	IC_50_: 10 *μ*mol/l for 72 h treatment	Colorectal cancer	BCL2L1, MYC, STAT3
*Anacardium occidentale* (cashews)	Cardanol monoene (CM) extracted from cashew nut shell liquid (CNSL)	IC_50_: 23.15 ± 2.42 *μ*M (24 h); 12.30 ± 1.67 *μ*M (48 h)	Melanoma	BAX, BCL2, TP53, CASP3, PARP1
*Annona muricata*	Ethyl acetate extract of *Annona muricata* leaves (EEAM)	IC_50_: 11.43 ± 1.87 *μ*g/ml (HT-29); 8.98 ± 1.24 *μ*g/ml (HCT-116) for 24 h treatment	Colon cancer	CASP3, CASP7, CASP8, CASP9, BAX, BCL2
*Arachis hypogaea* (peanuts)	Resveratrol	IC_50_: 46.81 ± 1.26 *μ*M (MCF-7); 53.02 ± 2.42 *μ*M (HeLa) for 72 h treatment	Breast cancer; cervical cancer	CDK4, CDKN1A, MAPK3, TP53
*Arachis hypogaea* (peanuts)	Peanut skin procyanidins (PSP)	IC_50_: 48.57 *μ*g/ml for 24 h treatment	Prostate cancer	BAX, BCL2, CASP3, TP53
*Basella rubra* (spinach)	Natural antioxidants (NAOs) from spinach extract	MIC: 3.2 mg/ml for 24 h treatment	Prostate cancer	CDK2, CDKN1A, E2F1, RB1, RBL1
Black pepper	Piperine	IC_50_: 54 ± 5 *μ*M to 126 ± 3 *μ*M for 72 h treatment	Colon cancer	AKT1, BIRC5, CCND1, CCND3, CDKN1A, CDKN1B, MAPK8
Black soybean	Black soybean extract	IC_50_: 3.69 mg/ml for 72 h treatment	Gastric cancer	BAX, BCL2, CASP3
*Brassica* spp. vegetables (cabbage, cauliflower, and brussels sprouts)	Indole-3-carbinol (I3C)	Maximum inhibition: 50 *μ*M for 24 h treatment	Acute myeloid leukemia (AML); leukemia	AKT1, BCL2, BIRC2, BIRC5, CCND1, MMP9, NFKB1, NOS2, PTGS2, TNF, TRAF1, XIAP
*Castanea* (chestnut)	Ethanol extracts of raw chestnut (RCE)	Maximum inhibition: 200 *μ*g/ml for 24 h treatment	Gastric cancer	ASP3, CASP7, CASP8, FASLG, HLA-DRB1,PARP1, TNFSF10, XIAP
*Citrus aurantium*	Flavonoids	IC_50_: 99 *μ*g/ml for 24 h treatment	Gastric cancer	CASP3, CCNB1, CDK1, PARP1
*Curcuma longa* (turmeric)	Curcumin	Maximum inhibition: 25 *μ*mol/l for 24 h treatment	Bladder cancer	NFKB1, PTGS2
*Curcuma longa* (turmeric)	Curcumin	IC_50_: 11 to 46 *μ*M (72 h)	Pancreatic cancer	CXCL8, NFKB1, PTGS2
*Curcuma longa* (turmeric)	Curcumin	Maximum inhibition: 50 *μ*M for 24 h treatment	Thyroid cancer	SMAD2, SMAD3, TGFB1
*Daphne genkwa*	Yuanhuadine	Maximum inhibition: 32 nM for 72 h treatment	Lung cancer	AKT1, CDK2, CDK4, CDKN1A, MYC
*Ipomoea batatas* (sweet potato)	Sporamin	Maximum inhibition: 100 *μ*M for 72 h treatment	Pancreatic cancer	BAX, BCL2, BCL2L1, NFKB1
*Juglans mandshruica* (Manchurian walnut)	Juglanin	IC_50_: 20.07 to 29.13 *μ*M for 24 h treatment	Breast cancer	BAD, BAX, BCL2, CASP3, CASP8, CASP9, CDC25C, CDK1, CDKN1B, CHEK2
*Juglans mandshruica* (Manchurian walnut)	Juglone	IC_50_: 8 *μ*M for 24 h treatment	Leukemia	AKT1, CASP3, MTOR, PIK3CD
Lebanese *Daucus carota* (wild carrot)	Daucus carota oil extract (DCOE)	IC_50_: 10.2 ± 0.90 to 19.1 ± 0.98 *μ*M for 48 h treatment	Skin Cancer	AKT1, BCl2, BAX
*Naematoloma sublateritium* (hazel mushroom)	Hexane fraction of N. sublateritium extract (HFNS)	IC_50_: 200 *μ*g/ml for 24 h treatment	Breast Cancer	JUN, MAPK14, MAPK8, MAPK9, MMP9, NFKB1, SERPINE1, TIMP2
*Pulsatilla koreana*	*Pulsatilla koreana* extract (PKE)	IC_50_: 120 to 140 *μ*g/ml for 24 h treatment	Thyroid cancer	BAX, BCL2, CASP3, PARP1
Red sorghum bran	3-Deoxyanthocyanidins	IC_50_: 300 *μ*g/ml for 24 h treatment	Breast Cancer	BCL2, TP53
*Sanguinaria canadensis* (bloodroot)	Sanguinarine	IC_50_: 1 *μ*M; maximum inhibition: 2 *μ*M for 24 h treatment	Oral cancer	AKT1, CASP3, CASP9, PIK3CD

MIC: minimum inhibitory concentration. IC50: half-maximal effective inhibitory concentration.

**Table 2:** Differentially expressed genes in diverse TCGA-derived cancers.

Gene	TCGA cancer type	Status
ATM	OV	Down
BAD	DLBC,THYM	Up
BAK1	PAAD	Up
BAX	DLBC,GBM,PAAD,TGCT,THYM	Up
BCL2	CESC,OV,UCS	Down
BCL2	DLBC,LAML,THYM	Up
BCL2L1	PAAD	Up
BIRC5	LAML	Down
BIRC5	BLCA,BRCA,CESC,COAD,DLBC,GBM,LIHC,LUAD,LUSC,OV,PAAD,READ,SKCM,STAD,THYM,UCEC,UCS	Up
CASP3	DLBC,GBM,PAAD,THYM	Up
CASP7	DLBC,THYM	Up
CASP9	OV	Down
CCNB1	LAML	Down
CCNB1	ACC,BLCA,BRCA,CESC,COAD,DLBC,GBM,LIHC,LUAD,LUSC,OV,PAAD,READ,SKCM,STAD,THYM,UCEC,UCS	Up
CCND1	LAML	Down
CCND1	COAD,DLBC,KIRC,LGG,OV,PAAD,READ,STAD,THCA,THYM	Up
CCND3	PAAD	Up
CDC25C	LAML	Down
CDC25C	CESC,COAD,DLBC,GBM,OV,READ,STAD,THYM,UCEC,UCS	Up
CDK1	LAML	Down
CDK1	ACC,BLCA,BRCA,CESC,COAD,DLBC,GBM,LIHC,LUAD,LUSC,OV,PAAD,READ,STAD,THYM,UCEC,UCS	Up
CDK2	DLBC,GBM,SKCM,THYM	Up
CDK4	DLBC,GBM,LGG,SARC,TGCT,THYM	Up
CDKN1A	OV	Down
CDKN1A	GBM	Up
CHEK2	DLBC,TGCT,THYM	Up
CXCL8	CESC,COAD,ESCA,GBM,HNSC,PAAD,READ,SKCM,STAD,UCEC	Up
E2F1	BLCA,BRCA,CESC,COAD,DLBC,LIHC,OV,PAAD,READ,THYM,UCEC,UCS	Up
HLA-DRB1	DLBC,GBM,LAML,LGG,OV,PAAD,STAD,TGCT,THYM	Up
JUN	SKCM	Down
MAPK1	LAML	Down
MAPK3	PAAD	Up
MAPK8	THYM	Up
MDM2	DLBC,SARC,THYM	Up
MMP2	ACC,CESC,OV,SKCM	Down
MMP2	DLBC,GBM,LAML,LGG,PAAD,THYM	Up
MMP9	THYM	Down
MMP9	BLCA,BRCA,CESC,COAD,ESCA,GBM,HNSC,OV,PAAD,READ,SKCM,STAD,TGCT,THCA,UCEC,UCS	Up
MTOR	THYM	Up
MYC	COAD,DLBC,GBM,LGG,READ,THYM	Up
NFKB1	THYM	Up
NOS2	COAD,READ	Up
PARP1	DLBC,THYM	Up
PIK3CD	SKCM	Up
PTGS2	LAML,PAAD	Up
RB1	GBM,THYM	Up
SERPINE1	KICH,OV	Down
SERPINE1	DLBC,ESCA,GBM,HNSC,PAAD	Up
SMAD3	UCEC	Down
SMAD3	THYM	Up
TGFB1	GBM,LGG,PAAD	Up
TIMP2	BLCA,CESC,OV,UCEC	Down
TIMP2	LAML,PAAD	Up
TNFSF10	KICH	Down
TNFSF10	CESC,LAML,PAAD,TGCT	Up
TP53	DLBC,GBM,LAML,LGG,THYM	Up
TRAF1	OV,UCS	Down
TRAF1	DLBC	Up
XIAP	THYM	Up

## Data Availability

The data used to support the findings of this study are available from the corresponding authors upon logical request.

## Abbreviations

ACC: Adrenocortical carcinoma
BLCA: Bladder urothelial carcinoma
BRCA: Breast-invasive carcinoma
CESC: Cervical squamous cell carcinoma and endocervical adenocarcinoma
COAD: Colon adenocarcinoma
DLBC: Lymphoid neoplasm diffuse large B-cell lymphoma
ESCA: Esophageal carcinoma
GBM: Glioblastoma multiforme
HNSC: Head and neck squamous cell carcinoma
KICH: Kidney chromophobe
KIRC: Kidney renal clear cell carcinoma
LAML: Acute myeloid leukemia
LGG: Brain lower-grade glioma
LIHC: Liver hepatocellular carcinoma
LUAD: Lung adenocarcinoma
LUSC: Lung squamous cell carcinoma
OV: Ovarian serous cystadenocarcinoma
PAAD: Pancreatic adenocarcinoma
READ: Rectum adenocarcinoma
SARC: Sarcoma
SKCM: Skin cutaneous melanoma
STAD: Stomach adenocarcinoma
TGCT: Testicular germ cell tumors
THCA: Thyroid carcinoma
THYM: Thymoma
UCEC: Uterine corpus endometrial carcinoma
UCS: Uterine carcinosarcoma.

## Supplementary Materials

Supplementary Materials Table S1: the class, natural source, active ingredient, the affected cancer type(s), target gene(s), gene expression status, cell line used in the experimental study, and corresponding PubMed link are listed.
