# Supplementary material for: Mining Natural Products with Anticancer Biological Activity through a Systems Biology Approach
Source: Oxid Med Cell Longev. 2021 Aug 12;2021:9993518. doi: 10.1155/2021/9993518 (PMC8376429; doi:10.1155/2021/9993518)
Supplement: Supplementary Materials — Table S1: the class, natural source, active ingredient, the affected cancer type(s), target gene(s), gene expression status, cell line used in the experimental study, and corresponding PubMed link are listed. [file 9993518.f1.docx]

| Source | Natural Product | Target Cancer | Target Gene | Status | Cell Line | Link Reference |
| --- | --- | --- | --- | --- | --- | --- |
| *Prunus armeniaca* (Armenicae semen) | Amygdalin | Colon cancer | ABCF2 | down | SNU-C4 | https://www.ncbi.nlm.nih.gov/pubmed/?term=16127745 |
| *Sanguinaria canadensis* (Bloodroot) | Sanguinarine | Oral cancer | AKT1 | down | KB | https://www.ncbi.nlm.nih.gov/pubmed/?term=27363951 |
| Teucrium alopecurus | D-limonene; B-Phellandrene; A-Cadinol; T-Muurolol; A-Bisabolol | Leukemia | BCL2L1 | down | KBM-5 | https://www.ncbi.nlm.nih.gov/pubmed/?term=28968948 |
| *Vitex agnus-castus* (Vitex) | Ethanol extract of Vitex | Gastric cancer | BCL2L1 | down | KATO-III | https://www.ncbi.nlm.nih.gov/pubmed/?term=15833280 |
| Lebanese *Daucus carota* (Wild carrot) | Daucus carota oil extract (DCOE) | Skin cancer | BCl2 | down | DMBA;TPA | https://www.ncbi.nlm.nih.gov/pubmed/?term=28073348 |
| *Vitex agnus-castus* (Vitex) | Ethanol extract of Vitex | Gastric cancer | BID | down | KATO-III | https://www.ncbi.nlm.nih.gov/pubmed/?term=15833280 |
| *Allium sativum* (Garlic) | N-benzyl-N-methyldecan-1-amine (NBNMA) | Leukemia | BIRC2 | down | U937 | https://www.ncbi.nlm.nih.gov/pubmed/?term=24859825 |
| *Teucrium alopecurus* | D-limonene; B-Phellandrene; A-Cadinol; T-Muurolol; A-Bisabolol | Leukemia | BIRC2 | down | KBM-5 | https://www.ncbi.nlm.nih.gov/pubmed/?term=28968948 |
| *Brassica* spp vegetables (cabbage, cauliflower, and brussels spouts) | Indole-3-carbinol (I3C) | Leukemia | BIRC2 | down | T-cell leukemia | https://www.ncbi.nlm.nih.gov/pubmed/?term=15811958 |
| *Brassica* spp vegetables (cabbage, cauliflower, and brussels spouts) | Indole-3-carbinol (I3C) | Acute myeloid leukemia (AML) | BIRC2 | down | KBM-5 | https://www.ncbi.nlm.nih.gov/pubmed/?term=15811958 |
| *Teucrium alopecurus* | D-limonene; B-Phellandrene; A-Cadinol; T-Muurolol; A-Bisabolol | Leukemia | BIRC3 | down | KBM-5 | https://www.ncbi.nlm.nih.gov/pubmed/?term=28968948 |
| *Teucrium alopecurus* | D-limonene; B-Phellandrene; A-Cadinol; T-Muurolol; A-Bisabolol | Leukemia | BIRC5 | down | KBM-5 | https://www.ncbi.nlm.nih.gov/pubmed/?term=28968948 |
| Black pepper | Piperine | Colon cancer | AKT1 | down | CACO-2;SW480;HT-29 | https://www.ncbi.nlm.nih.gov/pubmed/?term=24819444 |
| Black pepper | Piperine | Colon cancer | BIRC5 | down | CACO-2;SW480;HT-29 | https://www.ncbi.nlm.nih.gov/pubmed/?term=24819444 |
| *Brassica* spp vegetables (cabbage, cauliflower, and brussels spouts) | Indole-3-carbinol (I3C) | Leukemia | BIRC5 | down | T-cell leukemia | https://www.ncbi.nlm.nih.gov/pubmed/?term=15811958 |
| *Brassica* spp vegetables (cabbage, cauliflower, and brussels spouts) | Indole-3-carbinol (I3C) | Acute myeloid leukemia (AML) | BIRC5 | down | KBM-5 | https://www.ncbi.nlm.nih.gov/pubmed/?term=15811958 |
| *Sanguinaria canadensis* (Bloodroot) | Sanguinarine | Oral cancer | CASP3 | up | KB | https://www.ncbi.nlm.nih.gov/pubmed/?term=27363951 |
| Black soybean | Flavanols; Phenolics acids; Anthocyanins; Isoflavones | Gastric cancer | CASP3 | up | AGS | https://www.ncbi.nlm.nih.gov/pubmed/?term=21462927 |
| *Allium hirtifolium* (Persian shallot) | 2-Methylpyridine-1-ium-1-sulfonate (MPS) | Breast cancer | CASP3 | up | MCF-7;MDA-MB-231 | https://www.ncbi.nlm.nih.gov/pubmed/?term=28624423 |
| Black Rice | Anthocyanin-rich extract of black rice (AEBR) | Breast cancer | CASP3 | up | MDA-MB-453 | https://www.ncbi.nlm.nih.gov/pubmed/?term=21058201 |
| *Anguilla japonica* (Eel) | Eel skin mucus (ESM) | Leukemia | CASP3 | up | K562 | https://www.ncbi.nlm.nih.gov/pubmed/?term=26090845 |
| *Holothuria edulis* (Sea cucumber) | ESC-AQ (An aqueous fraction of the edible sea cucumber) | Leukemia | CASP3 | up | HL-60 | https://www.ncbi.nlm.nih.gov/pubmed/?term=23561113 |
| *Vitex agnus-castus* (Vitex) | Ethanol extract of Vitex | Gastric cancer | CASP3 | up | KATO-III | https://www.ncbi.nlm.nih.gov/pubmed/?term=15833280 |
| *Cucurbita moschata* (Pumpkin) | Cucurmosin | Pancreatic cancer | AKT1 | down | BxPC-3 | https://www.ncbi.nlm.nih.gov/pubmed/?term=22139427 |
| Camel’s milk | Lactoferrin | Breast cancer | CASP3 | up | BT-474 | https://www.ncbi.nlm.nih.gov/pubmed/?term=26434890 |
| *Capparis spinosa* (Caperbush) | N-butanol extract of Capparis spinosa (CSBE) | Gastric cancer | CASP3 | up | SGC-7901 | https://www.ncbi.nlm.nih.gov/pubmed/?term=26668648 |
| *Arachis hypogaea* (Peanuts) | Peanut skin procyanidins (PSP) | Prostate cancer | CASP3 | up | DU145 | https://www.ncbi.nlm.nih.gov/pubmed/?term=29654773 |
| Red pepper | Capsaicin (trans-8-methyl-N-vanillyl-6-nonenamide) | Leukemia | CASP3 | up | HL-60 | https://www.ncbi.nlm.nih.gov/pubmed/?term=16827131 |
| *Juglans mandshurica* (Walnut) | Juglanin | Breast cancer | CASP3 | up | MDA-MB-231;SKBR3;BT474;MCF-7 | https://www.ncbi.nlm.nih.gov/pubmed/?term=27899257 |
| *Portulaca* oleracea | Portulacerebroside A (PCA) | Leukemia | CASP3 | up | HL-60 | https://www.ncbi.nlm.nih.gov/pubmed/?term=26823708 |
| *Camellia sinensis* (Green tea) | Quercetin | Leukemia | CASP3 | up | HL-60 | https://www.ncbi.nlm.nih.gov/pubmed/?term=29472583 |
| *Annona muricata* | Ethyl acetate extract of Annona muricata leaves (EEAM) | Colon cancer | CASP3 | up | HT-29;HTC-116 | https://www.ncbi.nlm.nih.gov/pubmed/?term=25195082 |
| *Rosmarinus ofﬁcinalis* (Rosemary) | Carnosic acid (CA) | Colon cancer | CASP3 | up | SW480;HT-29;HCT116 | https://www.ncbi.nlm.nih.gov/pubmed/?term=26152521 |
| *Citrus aurantium* | Flavonioids | Gastric cancer | CASP3 | up | RPMI-1640 | https://www.ncbi.nlm.nih.gov/pubmed/?term=22194772 |
| Shallot | Flavonoid Isoliquiritigenin (ISL) | Cervical cancer | ATM | up | HeLa | https://www.ncbi.nlm.nih.gov/pubmed/?term=19536869 |
| *Punica granatum* L. (Pomegranate) | Pomegranate peel extract (PoPx); Punicalagin; Ellagic acid | Prostate cancer | CASP3 | up | DU145;PC3 | https://www.ncbi.nlm.nih.gov/pubmed/?term=28724216 |
| *Juglans mandshruica* (Manchurian walnut) | Juglone | Leukemia | CASP3 | up | HL-60 | https://www.ncbi.nlm.nih.gov/pubmed/?term=22266044 |
| *Anacardium occidentale* (Cashews) | Cardanol monoene (CM) extracted from cashew nut shell liquid (CNSL) | Melanoma | CASP3 | up | M14 | https://www.ncbi.nlm.nih.gov/pubmed/?term=28627168 |
| *Coix lachryma-jobi* (Adlay seed) | CP-1 polysaccharide | Lung cancer | CASP3 | up | A549 | https://www.ncbi.nlm.nih.gov/pubmed/?term=23200838 |
| *Litchi chinensis* (Litchi) | Litchi seed ethanol extract (LCSP) | Colon cancer | CASP3 | up | Colo320DM;SW480 | https://www.ncbi.nlm.nih.gov/pubmed/?term=23093841 |
| *Foeniculum vulgare* (Fennel) | Chloroform fraction of fennel (CFF) | Breast cancer | CASP3 | up | MCF-7 | https://www.ncbi.nlm.nih.gov/pubmed/?term=29474902 |
| *Ocimum basilicum*  (Sweet basil) | Lupeol epoxide | Breast cancer | CASP3 | up | MCF-7;MDA-MB-231 | https://www.ncbi.nlm.nih.gov/pubmed/?term=25548920 |
| *Corylus avellana* (Hazelnut) | Methanol hazelnut shells extract | Cervical cancer | CASP3 | up | HeLa | https://www.ncbi.nlm.nih.gov/pubmed/?term=28208804 |
| Anchovy (anchovy sause) | Ηydrophobic peptide fraction of the anchovy sauce (Aob) | Lymphoma | CASP3 | up | U937 | https://www.ncbi.nlm.nih.gov/pubmed/?term=15033760 |
| *Semecarpus anacardium* (Marking nut) | Semecarpus anacardium nut extract (SA) | Breast cancer | CASP3 | up | T47D | https://www.ncbi.nlm.nih.gov/pubmed/?term=17572113 |
| Aegle marmelos | Βeta caryophyllene and caryophyllene oxide fractions of Aegle marmelos extract | Lymphoma | ATM | up | IMR-32 | https://www.ncbi.nlm.nih.gov/pubmed/?term=24484210 |
| *Angelica sinensis* | Acetone extract (AE-AS) | Lung cancer | CASP3 | up | A549 | https://www.ncbi.nlm.nih.gov/pubmed/?term=15261763 |
| *Persea declinata* (Bl.) | Persea declinata (Bl.) Kosterm bark methanolic crude extract (PDM) | Breast cancer | CASP3 | up | MCF-7 | https://www.ncbi.nlm.nih.gov/pubmed/?term=24808916 |
| *Albizzia julibrissin* (Leguminosae) | Methanol extract of Albizzia julibrissin | Leukemia | CASP3 | up | Jurkat T | https://www.ncbi.nlm.nih.gov/pubmed/?term=16533581 |
| *Allium sativum* (Garlic) | N-benzyl-N-methyldecan-1-amine (NBNMA) | Leukemia | CASP3 | up | U937 | https://www.ncbi.nlm.nih.gov/pubmed/?term=24859825 |
| *Pulsatilla koreana* | Pulsatilla koreana extract (PKE) | Thyroid cancer | CASP3 | up | 8505c | https://www.ncbi.nlm.nih.gov/pubmed/?term=23135100 |
| *Castanea* (Chestnut) | Ethanol extracts of raw chestnut (RCE) | Gastric cancer | CASP3 | up | AGS | https://www.ncbi.nlm.nih.gov/pubmed/?term=21779520 |
| *Psidium guajava* (Guava) | Psidium gujava L. extracts (PE) | Prostate cancer | CASP3 | up | PZHPV-7;DU-145 | https://www.ncbi.nlm.nih.gov/pubmed/?term=17571972 |
| *Dipsacus asperoides* | Akebia saponin | Gastric cancer | CASP3 | down | AGS | https://www.ncbi.nlm.nih.gov/pubmed/?term=23850994 |
| *Musa paradisiaca* (Banana) | Methanol extract of Musa paradisiaca inflorescence (PIMET) | Colon cancer | CASP3 | up | HT29 | https://www.ncbi.nlm.nih.gov/pubmed/?term=29243757 |
| *Persea declinata* (Bl.) | Persea declinata (Bl.) Kosterm bark methanolic crude extract (PDM) | Breast cancer | CASP7 | up | MCF-7 | https://www.ncbi.nlm.nih.gov/pubmed/?term=24808916 |
| *Aegle marmelos* | Βeta caryophyllene and caryophyllene oxide fractions of Aegle marmelos extract | Neuroblastoma | ATM | up | IMR-32 | https://www.ncbi.nlm.nih.gov/pubmed/?term=24484210 |
| *Annona muricata* | Ethyl acetate extract of Annona muricata leaves (EEAM) | Colon cancer | CASP7 | up | HT-29;HTC-116 | https://www.ncbi.nlm.nih.gov/pubmed/?term=25195082 |
| *Castanea* (Chestnut) | Ethanol extracts of raw chestnut (RCE) | Gastric cancer | CASP7 | up | AGS | https://www.ncbi.nlm.nih.gov/pubmed/?term=21779520 |
| *Juglans mandshurica* (Walnut) | Juglanin | Breast cancer | CASP8 | up | MDA-MB-231;SKBR3;BT474;MCF-7 | https://www.ncbi.nlm.nih.gov/pubmed/?term=27899257 |
| Black caraway | Thymoquinone (TQ) | Breast cancer | CASP8 | up | MDA-MB-468;MDA-MB-231 | https://www.ncbi.nlm.nih.gov/pubmed/?term=24579801 |
| *Allium sativum* (Garlic) | N-benzyl-N-methyldecan-1-amine (NBNMA) | Leukemia | CASP8 | up | U937 | https://www.ncbi.nlm.nih.gov/pubmed/?term=24859825 |
| *Aegle marmelos* | Βeta caryophyllene and caryophyllene oxide fractions of Aegle marmelos extract | Lymphoma | CASP8 | up | IMR-32 | https://www.ncbi.nlm.nih.gov/pubmed/?term=24484210 |
| *Annona muricata* | Ethyl acetate extract of Annona muricata leaves (EEAM) | Colon cancer | CASP8 | up | HT-29;HTC-116 | https://www.ncbi.nlm.nih.gov/pubmed/?term=25195082 |
| *Vitex agnus-castus* (Vitex) | Ethanol extract of Vitex | Gastric cancer | CASP8 | up | KATO-III | https://www.ncbi.nlm.nih.gov/pubmed/?term=15833280 |
| *Castanea* (Chestnut) | Ethanol extracts of raw chestnut (RCE) | Gastric cancer | CASP8 | up | AGS | https://www.ncbi.nlm.nih.gov/pubmed/?term=21779520 |
| *Aegle marmelos* | Βeta caryophyllene and caryophyllene oxide fractions of Aegle marmelos extract | Neuroblastoma | CASP8 | up | IMR-32 | https://www.ncbi.nlm.nih.gov/pubmed/?term=24484210 |
| *Musa paradisiaca* (Banana) | Methanol extract of Musa paradisiaca inflorescence (PIMET) | Colon cancer | ATP8A2 | down | HT29 | https://www.ncbi.nlm.nih.gov/pubmed/?term=29243757 |
| Anchovy (anchovy sause) | Ηydrophobic peptide fraction of the anchovy sauce (Aob) | Lymphoma | CASP8 | up | U937 | https://www.ncbi.nlm.nih.gov/pubmed/?term=15033760 |
| *Foeniculum vulgare* (Fennel) | Chloroform fraction of fennel (CFF) | Breast cancer | CASP9 | up | MCF-7 | https://www.ncbi.nlm.nih.gov/pubmed/?term=29474902 |
| *Aegle marmelos* | Βeta caryophyllene and caryophyllene oxide fractions of Aegle marmelos extract | Neuroblastoma | CASP9 | up | IMR-32 | https://www.ncbi.nlm.nih.gov/pubmed/?term=24484210 |
| *Semecarpus anacardium* (Marking nut) | Semecarpus anacardium nut extract (SA) | Breast cancer | CASP9 | up | T47D | https://www.ncbi.nlm.nih.gov/pubmed/?term=17572113 |
| *Allium hirtifolium* (Persian shallot) | 2-Methylpyridine-1-ium-1-sulfonate (MPS) | Breast cancer | CASP9 | up | MCF-7;MDA-MB-231 | https://www.ncbi.nlm.nih.gov/pubmed/?term=28624423 |
| Shallot | Flavonoid Isoliquiritigenin (ISL) | Cervical cancer | CASP9 | up | HeLa | https://www.ncbi.nlm.nih.gov/pubmed/?term=19536869 |
| *Vitex agnus-castus* (Vitex) | Ethanol extract of Vitex | Gastric cancer | CASP9 | up | KATO-III | https://www.ncbi.nlm.nih.gov/pubmed/?term=15833280 |
| *Portulaca oleracea* | Portulacerebroside A (PCA) | Leukemia | CASP9 | up | HL-60 | https://www.ncbi.nlm.nih.gov/pubmed/?term=26823708 |
| *Juglans mandshurica* (Walnut) | Juglanin | Breast cancer | CASP9 | up | MDA-MB-231;SKBR3;BT474;MCF-7 | https://www.ncbi.nlm.nih.gov/pubmed/?term=27899257 |
| *Anguilla japonica* (Eel) | Eel skin mucus (ESM) | Leukemia | CASP9 | up | K562 | https://www.ncbi.nlm.nih.gov/pubmed/?term=26090845 |
| *Juglans mandshurica* (Walnut) | Juglanin | Breast cancer | BAD | up | MDA-MB-231;SKBR3;BT474;MCF-7 | https://www.ncbi.nlm.nih.gov/pubmed/?term=27899257 |
| Black caraway | Thymoquinone (TQ) | Breast cancer | CASP9 | up | MDA-MB-468;MDA-MB-231 | https://www.ncbi.nlm.nih.gov/pubmed/?term=24579801 |
| *Aegle marmelos* | Βeta caryophyllene and caryophyllene oxide fractions of Aegle marmelos extract | Lymphoma | CASP9 | up | IMR-32 | https://www.ncbi.nlm.nih.gov/pubmed/?term=24484210 |
| *Coix lachryma-jobi* (Adlay seed) | CP-1 polysaccharide | Lung cancer | CASP9 | up | A549 | https://www.ncbi.nlm.nih.gov/pubmed/?term=23200838 |
| Black Rice | Anthocyanin-rich extract of black rice (AEBR) | Breast cancer | CASP9 | up | MDA-MB-453 | https://www.ncbi.nlm.nih.gov/pubmed/?term=21058201 |
| *Rosmarinus ofﬁcinalis* (Rosemary) | Carnosic acid (CA) | Colon cancer | CASP9 | up | SW480;HT-29;HCT116 | https://www.ncbi.nlm.nih.gov/pubmed/?term=26152521 |
| *Allium sativum* (Garlic) | N-benzyl-N-methyldecan-1-amine (NBNMA) | Leukemia | CASP9 | up | U937 | https://www.ncbi.nlm.nih.gov/pubmed/?term=24859825 |
| *Albizzia julibrissin* (Leguminosae) | Methanol extract of Albizzia julibrissin | Leukemia | CASP9 | up | Jurkat T | https://www.ncbi.nlm.nih.gov/pubmed/?term=16533581 |
| *Cucurbita moschata* (Pumpkin) | Cucurmosin | Pancreatic cancer | CASP9 | up | BxPC-3 | https://www.ncbi.nlm.nih.gov/pubmed/?term=22139427 |
| *Annona muricata* | Ethyl acetate extract of Annona muricata leaves (EEAM) | Colon cancer | CASP9 | up | HT-29;HTC-116 | https://www.ncbi.nlm.nih.gov/pubmed/?term=25195082 |
| *Juglans mandshruica* (Manchurian walnut) | Juglone | Leukemia | CASP9 | up | HL-60 | https://www.ncbi.nlm.nih.gov/pubmed/?term=22266044 |
| *Vitex agnus-castus* (Vitex) | Ethanol extract of Vitex | Gastric cancer | BAD | up | KATO-III | https://www.ncbi.nlm.nih.gov/pubmed/?term=15833280 |
| *Angelica sinensis* | Acetone extract (AE-AS) | Lung cancer | CASP9 | up | A549 | https://www.ncbi.nlm.nih.gov/pubmed/?term=15261763 |
| *Capparis spinosa* (Caperbush) | N-butanol extract of Capparis spinosa (CSBE) | Gastric cancer | CASP9 | up | SGC-7901 | https://www.ncbi.nlm.nih.gov/pubmed/?term=26668648 |
| *Sanguinaria canadensis* (Bloodroot) | Sanguinarine | Oral cancer | CASP9 | up | KB | https://www.ncbi.nlm.nih.gov/pubmed/?term=27363951 |
| *Crocus sativus* (Saffron) | Saffron extract (SE); Crocin | Prostate cancer | CCNB1 | down | LAPC-4;PC3 | https://www.ncbi.nlm.nih.gov/pubmed/?term=23909737 |
| *Silybum marianum* (Thistle plant) | Silibinin | Colon cancer | CCNB1 | down | Fet;Geo;HCT116 | https://www.ncbi.nlm.nih.gov/pubmed/?term=17950073 |
| *Citrus aurantium* | Flavonioids | Gastric cancer | CCNB1 | down | RPMI-1640 | https://www.ncbi.nlm.nih.gov/pubmed/?term=22194772 |
| *Strychnos nux-vomica* (Loganiaceae) | Water extract of Strychni Semen (ESS) | Gastric cancer | CCNB1 | down | RPMI-1640 | https://www.ncbi.nlm.nih.gov/pubmed/?term=18446845 |
| *Litchi chinensis* (Litchi) | Litchi seed ethanol extract (LCSP) | Colon cancer | CCNB1 | down | Colo320DM;SW480 | https://www.ncbi.nlm.nih.gov/pubmed/?term=23093841 |
| *Litchi chinensis* (Litchi) | Litchi seed ethanol extract (LCSP) | Colon cancer | CCND1 | down | Colo320DM;SW480 | https://www.ncbi.nlm.nih.gov/pubmed/?term=23093841 |
| *Ribes hirtellum* (Gooseberry) | Polyphenolic compounds | Prostate cancer | CCND1 | down | PC-3 | https://www.ncbi.nlm.nih.gov/pubmed/?term=17465224 |
| *Allium sativum* (Garlic) | N-benzyl-N-methyldecan-1-amine (NBNMA) | Leukemia | BAD | up | U937 | https://www.ncbi.nlm.nih.gov/pubmed/?term=24859825 |
| *Silybum marianum* (Thistle plant) | Silibinin | Colon cancer | CCND1 | down | Fet;Geo;HCT116 | https://www.ncbi.nlm.nih.gov/pubmed/?term=17950073 |
| *Allium hirtifolium* (Persian shallot) | 2-Methylpyridine-1-ium-1-sulfonate (MPS) | Breast cancer | CCND1 | down | MCF-7;MDA-MB-231 | https://www.ncbi.nlm.nih.gov/pubmed/?term=28624423 |
| Black pepper | Piperine | Colon cancer | CCND1 | down | CACO-2;SW480;HT-29 | https://www.ncbi.nlm.nih.gov/pubmed/?term=24819444 |
| *Vaccinium macrocarpon* (Cranberry) | Polyphenolic compounds | Prostate cancer | CCND1 | down | PC-3 | https://www.ncbi.nlm.nih.gov/pubmed/?term=17465224 |
| *Crocus sativus* (Saffron) | Saffron extract (SE); Crocin | Prostate cancer | CCND1 | down | LAPC-4;PC3 | https://www.ncbi.nlm.nih.gov/pubmed/?term=23909737 |
| *Carya cathayensis* (Chinese hickory) | E2S ((E)-3-[(2S,3R)-2,3-dihydro-2-(4'-hydroxy-3'-methoxyphenyl)-3-hydroxymethyl-7-methoxy-1-benzo[b]furan-5-yl]-2-propenal) | Colorectal cancer | CCND1 | down | HCT116;HT29;SW480;LoVo | https://www.ncbi.nlm.nih.gov/pubmed/?term=24218372 |
| *Hippophae rhamnoides* (Sea buckthorn) | Polyphenolic compounds | Prostate cancer | CCND1 | down | PC-3 | https://www.ncbi.nlm.nih.gov/pubmed/?term=17465224 |
| *Brassica* spp vegetables (cabbage, cauliflower, and brussels spouts) | Indole-3-carbinol (I3C) | Leukemia | CCND1 | down | T-cell leukemia | https://www.ncbi.nlm.nih.gov/pubmed/?term=15811958 |
| *Brassica* spp vegetables (cabbage, cauliflower, and brussels spouts) | Indole-3-carbinol (I3C) | Acute myeloid leukemia (AML) | CCND1 | down | KBM-5 | https://www.ncbi.nlm.nih.gov/pubmed/?term=15811958 |
| Red pepper | Capsaicin (trans-8-methyl-N-vanillyl-6-nonenamide) | Leukemia | CCND3 | down | HL-60 | https://www.ncbi.nlm.nih.gov/pubmed/?term=16827131 |
| Lebanese *Daucus carota* (Wild carrot) | Daucus carota oil extract (DCOE) | Skin cancer | AKT1 | down | DMBA;TPA | https://www.ncbi.nlm.nih.gov/pubmed/?term=28073348 |
| *Aegle marmelos* | Βeta caryophyllene and caryophyllene oxide fractions of Aegle marmelos extract | Neuroblastoma | BAK1 | up | IMR-32 | https://www.ncbi.nlm.nih.gov/pubmed/?term=24484210 |
| Black pepper | Piperine | Colon cancer | CCND3 | down | CACO-2;SW480;HT-29 | https://www.ncbi.nlm.nih.gov/pubmed/?term=24819444 |
| *Hippophae rhamnoides* (Sea buckthorn) | Polyphenolic compounds | Prostate cancer | CCND3 | down | PC-3 | https://www.ncbi.nlm.nih.gov/pubmed/?term=17465224 |
| *Vaccinium macrocarpon* (Cranberry) | Polyphenolic compounds | Prostate cancer | CCND3 | down | PC-3 | https://www.ncbi.nlm.nih.gov/pubmed/?term=17465224 |
| *Ribes hirtellum* (Gooseberry) | Polyphenolic compounds | Prostate cancer | CCND3 | down | PC-3 | https://www.ncbi.nlm.nih.gov/pubmed/?term=17465224 |
| Red pepper | Capsaicin (trans-8-methyl-N-vanillyl-6-nonenamide) | Leukemia | CCNE1 | down | HL-60 | https://www.ncbi.nlm.nih.gov/pubmed/?term=16827131 |
| *Juglans mandshurica* (Walnut) | Juglanin | Breast cancer | CDC25C | down | MDA-MB-231;SKBR3;BT474;MCF-7 | https://www.ncbi.nlm.nih.gov/pubmed/?term=27899257 |
| Shallot | Flavonoid Isoliquiritigenin (ISL) | Cervical cancer | CDC25C | down | HeLa | https://www.ncbi.nlm.nih.gov/pubmed/?term=19536869 |
| *Citrus aurantium* | Flavonioids | Gastric cancer | CDK1 | down | RPMI-1640 | https://www.ncbi.nlm.nih.gov/pubmed/?term=22194772 |
| *Asiasari radix* | Ethanol extract of Asiasari radix (EEAR) | Colon cancer | CDK1 | down | HCT-116 | https://www.ncbi.nlm.nih.gov/pubmed/?term=23255939 |
| Red pepper | Capsaicin (trans-8-methyl-N-vanillyl-6-nonenamide) | Leukemia | CDK1 | down | HL-60 | https://www.ncbi.nlm.nih.gov/pubmed/?term=16827131 |
| Shallot | Flavonoid Isoliquiritigenin (ISL) | Cervical cancer | BAK1 | up | HeLa | https://www.ncbi.nlm.nih.gov/pubmed/?term=19536869 |
| *Allium sativum* (Garlic) | N-benzyl-N-methyldecan-1-amine (NBNMA) | Leukemia | CDK1 | down | U937 | https://www.ncbi.nlm.nih.gov/pubmed/?term=24859825 |
| *Juglans mandshurica* (Walnut) | Juglanin | Breast cancer | CDK1 | down | MDA-MB-231;SKBR3;BT474;MCF-7 | https://www.ncbi.nlm.nih.gov/pubmed/?term=27899257 |
| *Strychnos nux-vomica* (Loganiaceae) | Water extract of Strychni Semen (ESS) | Gastric cancer | CDK1 | up | RPMI-1640 | https://www.ncbi.nlm.nih.gov/pubmed/?term=18446845 |
| Shallot | Flavonoid Isoliquiritigenin (ISL) | Cervical cancer | CDK1 | down | HeLa | https://www.ncbi.nlm.nih.gov/pubmed/?term=19536869 |
| *Strychnos nux-vomica* (Loganiaceae) | Water extract of Strychni Semen (ESS) | Gastric cancer | CDK2 | down | RPMI-1640 | https://www.ncbi.nlm.nih.gov/pubmed/?term=18446845 |
| *Silybum marianum* (Thistle plant) | Silibinin | Colon cancer | CDK2 | down | Fet;Geo;HCT116 | https://www.ncbi.nlm.nih.gov/pubmed/?term=17950073 |
| Celery | Celery seed extract (CSE) | Gastric cancer | CDK2 | down | BGC-823 | https://www.ncbi.nlm.nih.gov/pubmed/?term=22320960 |
| *Daphne genkwa* | Yuanhuadine | Lung cancer | CDK2 | down | A549 | https://www.ncbi.nlm.nih.gov/pubmed/?term=21916433 |
| *Basella rubra* (Spinach) | Natural antioxidants (NAOs) from spinach extract | Prostate cancer | CDK2 | down | PC3 | https://www.ncbi.nlm.nih.gov/pubmed/?term=15327971 |
| *Allium sativum* (Garlic) | N-benzyl-N-methyldecan-1-amine (NBNMA) | Leukemia | CDK2 | down | U937 | https://www.ncbi.nlm.nih.gov/pubmed/?term=24859825 |
| *Aegle marmelos* | Βeta caryophyllene and caryophyllene oxide fractions of Aegle marmelos extract | Lymphoma | BAK1 | up | IMR-32 | https://www.ncbi.nlm.nih.gov/pubmed/?term=24484210 |
| *Vaccinium macrocarpon* (Cranberry) | Polyphenolic compounds | Prostate cancer | CDK4 | down | PC-3 | https://www.ncbi.nlm.nih.gov/pubmed/?term=17465224 |
| *Angelica sinensis* | Acetone extract (AE-AS) | Lung cancer | CDK4 | down | A549 | https://www.ncbi.nlm.nih.gov/pubmed/?term=15261763 |
| *Hippophae rhamnoides* (Sea buckthorn) | Polyphenolic compounds | Prostate cancer | CDK4 | down | PC-3 | https://www.ncbi.nlm.nih.gov/pubmed/?term=17465224 |
| *Arachis hypogaea* (Peanuts) | Resveratrol | Breast cancer | CDK4 | down | MCF-7 | https://www.ncbi.nlm.nih.gov/pubmed/?term=27588384 |
| *Daphne genkwa* | Yuanhuadine | Lung cancer | CDK4 | down | A549 | https://www.ncbi.nlm.nih.gov/pubmed/?term=21916433 |
| *Carya cathayensis* (Chinese hickory) | E2S ((E)-3-[(2S,3R)-2,3-dihydro-2-(4'-hydroxy-3'-methoxyphenyl)-3-hydroxymethyl-7-methoxy-1-benzo[b]furan-5-yl]-2-propenal) | Colorectal cancer | CDK4 | down | HCT116;HT29;SW480;LoVo | https://www.ncbi.nlm.nih.gov/pubmed/?term=24218372 |
| *Ribes hirtellum* (Gooseberry) | Polyphenolic compounds | Prostate cancer | CDK4 | down | PC-3 | https://www.ncbi.nlm.nih.gov/pubmed/?term=17465224 |
| *Allium hirtifolium* (Persian shallot) | 2-Methylpyridine-1-ium-1-sulfonate (MPS) | Breast cancer | CDK4 | down | MCF-7;MDA-MB-231 | https://www.ncbi.nlm.nih.gov/pubmed/?term=28624423 |
| *Arachis hypogaea* (Peanuts) | Resveratrol | Cervical cancer | CDK4 | down | HeLa | https://www.ncbi.nlm.nih.gov/pubmed/?term=27588384 |
| *Vaccinium macrocarpon* (Cranberry) | Polyphenolic compounds | Prostate cancer | CDK6 | down | PC-3 | https://www.ncbi.nlm.nih.gov/pubmed/?term=17465224 |
| *Allium hirtifolium* (Persian shallot) | 2-Methylpyridine-1-ium-1-sulfonate (MPS) | Breast cancer | BAX | up | MCF-7;MDA-MB-231 | https://www.ncbi.nlm.nih.gov/pubmed/?term=28624423 |
| *Ribes hirtellum* (Gooseberry) | Polyphenolic compounds | Prostate cancer | CDK6 | down | PC-3 | https://www.ncbi.nlm.nih.gov/pubmed/?term=17465224 |
| *Hippophae rhamnoides* (Sea buckthorn) | Polyphenolic compounds | Prostate cancer | CDK6 | down | PC-3 | https://www.ncbi.nlm.nih.gov/pubmed/?term=17465224 |
| *Silybum marianum* (Thistle plant) | Silibinin | Colon cancer | CDKN1A | up | Fet;Geo;HCT116 | https://www.ncbi.nlm.nih.gov/pubmed/?term=17950073 |
| *Arachis hypogaea* (Peanuts) | Resveratrol | Cervical cancer | CDKN1A | up | HeLa | https://www.ncbi.nlm.nih.gov/pubmed/?term=27588384 |
| *Crocus sativus* (Saffron) | Saffron extract (SE); Crocin | Prostate cancer | CDKN1A | up | LAPC-4;PC3 | https://www.ncbi.nlm.nih.gov/pubmed/?term=23909737 |
| *Basella rubra* (Spinach) | Natural antioxidants (NAOs) from spinach extract | Prostate cancer | CDKN1A | up | PC3 | https://www.ncbi.nlm.nih.gov/pubmed/?term=15327971 |
| *Daphne genkwa* | Yuanhuadine | Lung cancer | CDKN1A | up | A549 | https://www.ncbi.nlm.nih.gov/pubmed/?term=21916433 |
| Shallot | Flavonoid Isoliquiritigenin (ISL) | Cervical cancer | CDKN1A | up | HeLa | https://www.ncbi.nlm.nih.gov/pubmed/?term=19536869 |
| *Allium hirtifolium* (Persian shallot) | 2-Methylpyridine-1-ium-1-sulfonate (MPS) | Breast cancer | CDKN1A | up | MCF-7;MDA-MB-231 | https://www.ncbi.nlm.nih.gov/pubmed/?term=28624423 |
| *Arachis hypogaea* (Peanuts) | Resveratrol | Breast cancer | CDKN1A | up | MCF-7 | https://www.ncbi.nlm.nih.gov/pubmed/?term=27588384 |
| Red pepper | Capsaicin (trans-8-methyl-N-vanillyl-6-nonenamide) | Leukemia | BAX | up | HL-60 | https://www.ncbi.nlm.nih.gov/pubmed/?term=16827131 |
| *Strychnos nux-vomica* (Loganiaceae) | Water extract of Strychni Semen (ESS) | Gastric cancer | CDKN1A | up | RPMI-1640 | https://www.ncbi.nlm.nih.gov/pubmed/?term=18446845 |
| Black pepper | Piperine | Colon cancer | CDKN1A | up | CACO-2;SW480;HT-29 | https://www.ncbi.nlm.nih.gov/pubmed/?term=24819444 |
| *Allium sativum* (Garlic) | N-benzyl-N-methyldecan-1-amine (NBNMA) | Leukemia | CDKN1A | up | U937 | https://www.ncbi.nlm.nih.gov/pubmed/?term=24859825 |
| *Crocus sativus* (Saffron) | Saffron extract (SE); Crocin | Prostate cancer | CDKN1B | up | LAPC-4;PC3 | https://www.ncbi.nlm.nih.gov/pubmed/?term=23909737 |
| *Juglans mandshurica* (Walnut) | Juglanin | Breast cancer | CDKN1B | up | MDA-MB-231;SKBR3;BT474;MCF-7 | https://www.ncbi.nlm.nih.gov/pubmed/?term=27899257 |
| *Silybum marianum* (Thistle plant) | Silibinin | Colon cancer | CDKN1B | up | Fet;Geo;HCT116 | https://www.ncbi.nlm.nih.gov/pubmed/?term=17950073 |
| *Allium hirtifolium* (Persian shallot) | 2-Methylpyridine-1-ium-1-sulfonate (MPS) | Breast cancer | CDKN1B | up | MCF-7;MDA-MB-231 | https://www.ncbi.nlm.nih.gov/pubmed/?term=28624423 |
| *Persea americana* (Avocado) | Carotenoids (zeaxanthin, α-carotene, and β-carotene); Lutein | Prostate cancer | CDKN1B | up | LNCaP;PC3 | https://www.ncbi.nlm.nih.gov/pubmed/?term=15629237 |
| Black pepper | Piperine | Colon cancer | CDKN1B | up | CACO-2;SW480;HT-29 | https://www.ncbi.nlm.nih.gov/pubmed/?term=24819444 |
| *Teucrium alopecurus* | D-limonene; B-Phellandrene; A-Cadinol; T-Muurolol; A-Bisabolol | Leukemia | CFLAR | down | KBM-5 | https://www.ncbi.nlm.nih.gov/pubmed/?term=28968948 |
| *Cinnamomum verum* (Cinnamon) | 2-methoxycinnamaldehyde (2-MCA) | Lung cancer | BAX | up | A549 | https://www.ncbi.nlm.nih.gov/pubmed/?term=26676220 |
| *Laurus nobilis* (Bay laurel) | Lauroside B (megastigmane glycoside) | Melanoma | CFLAR | down | A375;WM115;SK-Mel-28 | https://www.ncbi.nlm.nih.gov/pubmed/?term=21188975 |
| *Juglans mandshurica* (Walnut) | Juglanin | Breast cancer | CHEK2 | up | MDA-MB-231;SKBR3;BT474;MCF-7 | https://www.ncbi.nlm.nih.gov/pubmed/?term=27899257 |
| *Malus pumila* (Apple) | Flavonoids from an apple extract (AE) | Colon cancer | CHST5 | up | HT-29 | https://www.ncbi.nlm.nih.gov/pubmed/?term=16369997 |
| *Malus pumila* (Apple) | Flavonoids from an apple extract (AE) | Colon cancer | CHST6 | up | HT-29 | https://www.ncbi.nlm.nih.gov/pubmed/?term=16369997 |
| *Malus pumila* (Apple) | Flavonoids from an apple extract (AE) | Colon cancer | CHST7 | up | HT-29 | https://www.ncbi.nlm.nih.gov/pubmed/?term=16369997 |
| *Carya cathayensis* (Chinese hickory) | E2S ((E)-3-[(2S,3R)-2,3-dihydro-2-(4'-hydroxy-3'-methoxyphenyl)-3-hydroxymethyl-7-methoxy-1-benzo[b]furan-5-yl]-2-propenal) | Colorectal cancer | CTNNB1 | down | HCT116;HT29;SW480;LoVo | https://www.ncbi.nlm.nih.gov/pubmed/?term=24218372 |
| *Curcuma longa* (Turmeric) | Cucumin | Pancreatic cancer | CXCL8 | down | BxPC-3;Capan-1;Capan-2;ASPC-1;HS766-T | https://www.ncbi.nlm.nih.gov/pubmed/?term=15476283 |
| *Basella rubra* (Spinach) | Natural antioxidants (NAOs) from spinach extract | Prostate cancer | E2F1 | down | PC3 | https://www.ncbi.nlm.nih.gov/pubmed/?term=15327971 |
| *Cucurbita moschata* (Pumpkin) | Cucurmosin | Pancreatic cancer | EGFR | down | BxPC-3 | https://www.ncbi.nlm.nih.gov/pubmed/?term=22139427 |
| Lebanese *Daucus carota* (Wild carrot) | Daucus carota oil extract (DCOE) | Skin cancer | EPHB2 | down | DMBA;TPA | https://www.ncbi.nlm.nih.gov/pubmed/?term=28073348 |
| *Aegle marmelos* | Βeta caryophyllene and caryophyllene oxide fractions of Aegle marmelos extract | Neuroblastoma | BAX | up | IMR-32 | https://www.ncbi.nlm.nih.gov/pubmed/?term=24484210 |
| *Allium sativum* (Garlic) | Allicin | Glioblastoma | EPHB2 | up | U87MG | https://www.ncbi.nlm.nih.gov/pubmed/?term=22552443 |
| *Ananas comosus* (Pineapple) | Bromelain | Colorectal cancer | EPHB2 | down | CACO-2;DLD-1 | https://www.ncbi.nlm.nih.gov/pubmed/?term=24123777 |
| *Malus pumila* (Apple) | Flavonoids from an apple extract (AE) | Colon cancer | EPHX1 | down | HT-29 | https://www.ncbi.nlm.nih.gov/pubmed/?term=16369997 |
| Yogurt/Cheese | Lactobacillus spp | Colon cancer | ERBB2 | down | HT-29 | https://www.ncbi.nlm.nih.gov/pubmed/?term=28683007 |
| Yogurt/Cheese | Lactobacillus spp | Colon cancer | ERBB3 | down | HT-29 | https://www.ncbi.nlm.nih.gov/pubmed/?term=28683007 |
| *Prunus armeniaca* (Armenicae semen) | Amygdalin | Colon cancer | EXO1 | down | SNU-C4 | https://www.ncbi.nlm.nih.gov/pubmed/?term=16127745 |
| Olive oil | Hydroxytyrosol (HT) | Colon cancer | FAS | down | SW620 | https://www.ncbi.nlm.nih.gov/pubmed/?term=21437031 |
| *Castanea* (Chestnut) | Ethanol extracts of raw chestnut (RCE) | Gastric cancer | FASLG | up | AGS | https://www.ncbi.nlm.nih.gov/pubmed/?term=21779520 |
| Powdered Milk | Lactobacillus spp | Colon cancer | FOS | up | ΗΤ-29;HCT116 | https://www.ncbi.nlm.nih.gov/pubmed/?term=25276792 |
| *Momordica charantia* (Bitter gourd ) | Fatty acids prepared from Bitter gourd oil (BGO-FFA) | Colon cancer | GADD45A | up | CACO-2 | https://www.ncbi.nlm.nih.gov/pubmed/?term=15961301 |
| *Rosmarinus ofﬁcinalis* (Rosemary) | Carnosic acid (CA) | Colon cancer | BAX | up | SW480;HT-29;HCT116 | https://www.ncbi.nlm.nih.gov/pubmed/?term=26152521 |
| *Malus pumila* (Apple) | Flavonoids from an apple extract (AE) | Colon cancer | GSTP1 | up | HT-29 | https://www.ncbi.nlm.nih.gov/pubmed/?term=16369997 |
| Black caraway | Thymoquinone (TQ) | Breast cancer | H2AX | up | MDA-MB-468;MDA-MB-231 | https://www.ncbi.nlm.nih.gov/pubmed/?term=24579801 |
| *Castanea* (Chestnut) | Ethanol extracts of raw chestnut (RCE) | Gastric cancer | HLA-DRB1 | up | AGS | https://www.ncbi.nlm.nih.gov/pubmed/?term=21779520 |
| Chinese red yeast rice | Lovastatin (LV) | Colon cancer | HMGCR | up | HCT-116;HT-29 | https://www.ncbi.nlm.nih.gov/pubmed/?term=17869085 |
| *Panax quinquefolius* (American ginseng ) | Ginsenoside Rh2 (GRh2) | Colon cancer | HRH3 | down | HCT116 | https://www.ncbi.nlm.nih.gov/pubmed/?term=27746693 |
| *Musa paradisiaca* (Banana) | Methanol extract of Musa paradisiaca inflorescence (PIMET) | Colon cancer | HSPA5 | up | HT29 | https://www.ncbi.nlm.nih.gov/pubmed/?term=29243757 |
| *Teucrium alopecurus* | D-limonene; B-Phellandrene; A-Cadinol; T-Muurolol; A-Bisabolol | Leukemia | ICAM1 | down | KBM-5 | https://www.ncbi.nlm.nih.gov/pubmed/?term=28968948 |
| Bovine milk | Lactoferrin | Lung cancer | IL10 | down | A549 | https://www.ncbi.nlm.nih.gov/pubmed/?term=23462173 |
| Powdered Milk | Lactobacillus spp | Colon cancer | IL1B | down | ΗΤ-29;HCT116 | https://www.ncbi.nlm.nih.gov/pubmed/?term=25276792 |
| Bovine milk | Lactoferrin | Lung cancer | IL4 | down | A549 | https://www.ncbi.nlm.nih.gov/pubmed/?term=23462173 |
| *Allium sativum* (Garlic) | N-benzyl-N-methyldecan-1-amine (NBNMA) | Leukemia | BAX | up | U937 | https://www.ncbi.nlm.nih.gov/pubmed/?term=24859825 |
| Bovine milk | Lactoferrin | Lung cancer | IL6 | down | A549 | https://www.ncbi.nlm.nih.gov/pubmed/?term=23462173 |
| *Naematoloma sublateritium* (Hazel mushroom) | hexane fraction of N. sublateritium extract (HFNS) | Breast cancer | JUN | down | MDA-MB‑231 | https://www.ncbi.nlm.nih.gov/pubmed/?term=24968898 |
| Powdered Milk | Lactobacillus spp | Colon cancer | JUN | up | ΗΤ-29;HCT116 | https://www.ncbi.nlm.nih.gov/pubmed/?term=25276792 |
| *Panax quinquefolius* (American ginseng ) | Ginsenoside Rh2 (GRh2) | Colon cancer | MAPK1 | down | HCT116 | https://www.ncbi.nlm.nih.gov/pubmed/?term=27746693 |
| *Allium sativum* (Garlic) | Allicin | Glioblastoma | MAPK1 | up | U87MG | https://www.ncbi.nlm.nih.gov/pubmed/?term=22552443 |
| *Portulaca oleracea* | Portulacerebroside A (PCA) | Leukemia | MAPK1 | down | HL-60 | https://www.ncbi.nlm.nih.gov/pubmed/?term=26823708 |
| *Actinidia callosa* var. callosa | Ethyl acetate fraction of Actinidia callosa var. callosa (EAAC) | Hepatoma | MAPK1 | down | SK-Hep1 | https://www.ncbi.nlm.nih.gov/pubmed/?term=29356905 |
| *Anguilla japonica* (Eel) | Eel skin mucus (ESM) | Leukemia | MAPK1 | up | K562 | https://www.ncbi.nlm.nih.gov/pubmed/?term=26090845 |
| *Dipsacus asperoides* | Akebia saponin | Gastric cancer | MAPK1 | down | AGS | https://www.ncbi.nlm.nih.gov/pubmed/?term=23850994 |
| *Naematoloma sublateritium*  (Hazel mushroom) | hexane fraction of N. sublateritium extract (HFNS) | Breast cancer | MAPK14 | down | MDA-MB‑231 | https://www.ncbi.nlm.nih.gov/pubmed/?term=24968898 |
| *Juglans mandshurica* (Walnut) | Juglanin | Breast cancer | BAX | up | MDA-MB-231;SKBR3;BT474;MCF-7 | https://www.ncbi.nlm.nih.gov/pubmed/?term=27899257 |
| *Anguilla japonica* (Eel) | Eel skin mucus (ESM) | Leukemia | MAPK14 | up | K562 | https://www.ncbi.nlm.nih.gov/pubmed/?term=26090845 |
| *Arachis hypogaea* (Peanuts) | Resveratrol | Cervical cancer | MAPK3 | down | HeLa | https://www.ncbi.nlm.nih.gov/pubmed/?term=27588384 |
| *Arachis hypogaea* (Peanuts) | Resveratrol | Breast cancer | MAPK3 | down | MCF-7 | https://www.ncbi.nlm.nih.gov/pubmed/?term=27588384 |
| *Anguilla japonica* (Eel) | Eel skin mucus (ESM) | Leukemia | MAPK3 | up | K562 | https://www.ncbi.nlm.nih.gov/pubmed/?term=26090845 |
| *Panax quinquefolius* (American ginseng ) | Ginsenoside Rh2 (GRh2) | Colon cancer | MAPK3 | down | HCT116 | https://www.ncbi.nlm.nih.gov/pubmed/?term=27746693 |
| *Portulaca oleracea* | Portulacerebroside A (PCA) | Leukemia | MAPK8 | down | HL-60 | https://www.ncbi.nlm.nih.gov/pubmed/?term=26823708 |
| *Naematoloma sublateritium*  (Hazel mushroom) | hexane fraction of N. sublateritium extract (HFNS) | Breast cancer | MAPK8 | down | MDA-MB‑231 | https://www.ncbi.nlm.nih.gov/pubmed/?term=24968898 |
| Black pepper | Piperine | Colon cancer | MAPK8 | up | CACO-2;SW480;HT-29 | https://www.ncbi.nlm.nih.gov/pubmed/?term=24819444 |
| *Naematoloma sublateritium*  (Hazel mushroom) | hexane fraction of N. sublateritium extract (HFNS) | Breast cancer | MAPK9 | down | MDA-MB‑231 | https://www.ncbi.nlm.nih.gov/pubmed/?term=24968898 |
| *Teucrium alopecurus* | D-limonene; B-Phellandrene; A-Cadinol; T-Muurolol; A-Bisabolol | Leukemia | MCL1 | down | KBM-5 | https://www.ncbi.nlm.nih.gov/pubmed/?term=28968948 |
| *Juglans mandshruica* (Manchurian walnut) | Juglone | Leukemia | AKT1 | down | HL-60 | https://www.ncbi.nlm.nih.gov/pubmed/?term=22266044 |
| Lebanese *Daucus carota* (Wild carrot) | Daucus carota oil extract (DCOE) | Skin cancer | BAX | up | DMBA;TPA | https://www.ncbi.nlm.nih.gov/pubmed/?term=28073348 |
| *Camellia sinensis*  (Green tea) | Quercetin | Leukemia | MCL1 | down | HL-60 | https://www.ncbi.nlm.nih.gov/pubmed/?term=29472583 |
| *Aegle marmelos* | Βeta caryophyllene and caryophyllene oxide fractions of Aegle marmelos extract | Neuroblastoma | MDM2 | down | IMR-32 | https://www.ncbi.nlm.nih.gov/pubmed/?term=24484210 |
| *Aegle marmelos* | Βeta caryophyllene and caryophyllene oxide fractions of Aegle marmelos extract | Lymphoma | MDM2 | down | IMR-32 | https://www.ncbi.nlm.nih.gov/pubmed/?term=24484210 |
| *Rosmarinus ofﬁcinalis* (Rosemary) | Carnosic acid (CA) | Colon cancer | MDM2 | down | SW480;HT-29;HCT116 | https://www.ncbi.nlm.nih.gov/pubmed/?term=26152521 |
| *Malus pumila* (Apple) | Flavonoids from an apple extract (AE) | Colon cancer | MGST2 | up | HT-29 | https://www.ncbi.nlm.nih.gov/pubmed/?term=16369997 |
| *Allium hirtifolium* (Persian shallot) | 2-Methylpyridine-1-ium-1-sulfonate (MPS) | Breast cancer | MMP2 | down | MCF-7;MDA-MB-231 | https://www.ncbi.nlm.nih.gov/pubmed/?term=28624423 |
| *Psidium guajava* (Guava) | Psidium gujava L. extracts (PE) | Prostate cancer | MMP2 | down | PZHPV-7;DU-145 | https://www.ncbi.nlm.nih.gov/pubmed/?term=17571972 |
| *Punica granatum* L. (Pomegranate) | Pomegranate peel extract (PoPx); Punicalagin; Ellagic acid | Prostate cancer | MMP2 | down | DU145;PC3 | https://www.ncbi.nlm.nih.gov/pubmed/?term=28724216 |
| *Actinidia callosa* var. callosa | Ethyl acetate fraction of Actinidia callosa var. callosa (EAAC) | Hepatoma | MMP2 | down | SK-Hep1 | https://www.ncbi.nlm.nih.gov/pubmed/?term=29356905 |
| *Vitis vinifera* (Grape) | Proanthocyanidin | Skin cancer | MMP2 | down | SCC12 | https://www.ncbi.nlm.nih.gov/pubmed/?term=28781636 |
| Black soybean | Flavanols; Phenolics acids; Anthocyanins; Isoflavones | Gastric cancer | BAX | up | AGS | https://www.ncbi.nlm.nih.gov/pubmed/?term=21462927 |
| Eriobotrya japonica (Korean mandolin) | Εthyl ethanoate (EtOAc) | Lung cancer | MMP2 | down | B16F19 | https://www.ncbi.nlm.nih.gov/pubmed/?term=21547674 |
| *Vitis coignetiae* | Anthocyanins | Lung cancer | MMP2 | down | A549 | https://www.ncbi.nlm.nih.gov/pubmed/?term=29172259 |
| Oregano/Thyme | Carvacrol | Colon cancer | MMP2 | down | HCT116;LovO | https://www.ncbi.nlm.nih.gov/pubmed/?term=26214321 |
| *Vitis coignetiae* | Anthocyanins | Lung cancer | MMP9 | down | A549 | https://www.ncbi.nlm.nih.gov/pubmed/?term=29172259 |
| *Naematoloma sublateritium*  (Hazel mushroom) | hexane fraction of N. sublateritium extract (HFNS) | Breast cancer | MMP9 | down | MDA-MB‑231 | https://www.ncbi.nlm.nih.gov/pubmed/?term=24968898 |
| *Allium hirtifolium* (Persian shallot) | 2-Methylpyridine-1-ium-1-sulfonate (MPS) | Breast cancer | MMP9 | down | MCF-7;MDA-MB-231 | https://www.ncbi.nlm.nih.gov/pubmed/?term=28624423 |
| *Vitis vinifera* (Grape) | Proanthocyanidin | Skin cancer | MMP9 | down | SCC12 | https://www.ncbi.nlm.nih.gov/pubmed/?term=28781636 |
| *Psidium guajava* (Guava) | Psidium gujava L. extracts (PE) | Prostate cancer | MMP9 | down | PZHPV-7;DU-145 | https://www.ncbi.nlm.nih.gov/pubmed/?term=17571972 |
| *Punica granatum* L. (Pomegranate) | Pomegranate peel extract (PoPx); Punicalagin; Ellagic acid | Prostate cancer | MMP9 | down | DU145;PC3 | https://www.ncbi.nlm.nih.gov/pubmed/?term=28724216 |
| Oregano/Thyme | Carvacrol | Colon cancer | MMP9 | down | HCT116;LovO | https://www.ncbi.nlm.nih.gov/pubmed/?term=26214321 |
| *Ocimum basilicum*  (Sweet basil) | Lupeol epoxide | Breast cancer | BAX | up | MCF-7;MDA-MB-231 | https://www.ncbi.nlm.nih.gov/pubmed/?term=25548920 |
| *Eriobotrya japonica* (Korean mandolin) | Εthyl ethanoate (EtOAc) | Lung cancer | MMP9 | down | B16F19 | https://www.ncbi.nlm.nih.gov/pubmed/?term=21547674 |
| *Actinidia callosa* var. callosa | Ethyl acetate fraction of Actinidia callosa var. callosa (EAAC) | Hepatoma | MMP9 | down | SK-Hep1 | https://www.ncbi.nlm.nih.gov/pubmed/?term=29356905 |
| *Teucrium alopecurus* | D-limonene; B-Phellandrene; A-Cadinol; T-Muurolol; A-Bisabolol | Leukemia | MMP9 | down | KBM-5 | https://www.ncbi.nlm.nih.gov/pubmed/?term=28968948 |
| *Brassica* spp vegetables (cabbage, cauliflower, and brussels spouts) | Indole-3-carbinol (I3C) | Leukemia | MMP9 | down | T-cell leukemia | https://www.ncbi.nlm.nih.gov/pubmed/?term=15811958 |
| *Brassica* spp vegetables (cabbage, cauliflower, and brussels spouts) | Indole-3-carbinol (I3C) | Acute myeloid leukemia (AML) | MMP9 | down | KBM-5 | https://www.ncbi.nlm.nih.gov/pubmed/?term=15811958 |
| *Prunus armeniaca* (Armenicae semen) | Amygdalin | Colon cancer | MRE11 | down | SNU-C4 | https://www.ncbi.nlm.nih.gov/pubmed/?term=16127745 |
| *Juglans mandshruica* (Manchurian walnut) | Juglone | Leukemia | MTOR | down | HL-60 | https://www.ncbi.nlm.nih.gov/pubmed/?term=22266044 |
| *Cucurbita moschata* (Pumpkin) | Cucurmosin | Pancreatic cancer | MTOR | down | BxPC-3 | https://www.ncbi.nlm.nih.gov/pubmed/?term=22139427 |
| *Prunus armeniaca* (Armenicae semen) | Amygdalin | Colon cancer | MTOR | down | SNU-C4 | https://www.ncbi.nlm.nih.gov/pubmed/?term=16127745 |
| *Aegle marmelos* | Βeta caryophyllene and caryophyllene oxide fractions of Aegle marmelos extract | Neuroblastoma | MYB | down | IMR-32 | https://www.ncbi.nlm.nih.gov/pubmed/?term=24484210 |
| *Aegle marmelos* | Βeta caryophyllene and caryophyllene oxide fractions of Aegle marmelos extract | Lymphoma | BAX | up | IMR-32 | https://www.ncbi.nlm.nih.gov/pubmed/?term=24484210 |
| *Aegle marmelos* | Βeta caryophyllene and caryophyllene oxide fractions of Aegle marmelos extract | Lymphoma | MYB | down | IMR-32 | https://www.ncbi.nlm.nih.gov/pubmed/?term=24484210 |
| *Aloe vera* (Barbados aloe) | Aloin (AL) | Colorectal cancer | MYC | down | Fet;Geo;HCT116 | https://www.ncbi.nlm.nih.gov/pubmed/?term=23848964 |
| Red *Panax ginseng* (Red Korean ginseng) | Korean red ginseng extract (KRGE) | Leukemia | MYC | down | NB4 | https://www.ncbi.nlm.nih.gov/pubmed/?term=24095829 |
| *Carya cathayensis* (Chinese hickory) | E2S ((E)-3-[(2S,3R)-2,3-dihydro-2-(4'-hydroxy-3'-methoxyphenyl)-3-hydroxymethyl-7-methoxy-1-benzo[b]furan-5-yl]-2-propenal) | Colorectal cancer | MYC | down | HCT116;HT29;SW480;LoVo | https://www.ncbi.nlm.nih.gov/pubmed/?term=24218372 |
| *Daphne genkwa* | Yuanhuadine | Lung cancer | MYC | down | A549 | https://www.ncbi.nlm.nih.gov/pubmed/?term=21916433 |
| *Carya cathayensis* (Chinese hickory) | E2S ((E)-3-[(2S,3R)-2,3-dihydro-2-(4'-hydroxy-3'-methoxyphenyl)-3-hydroxymethyl-7-methoxy-1-benzo[b]furan-5-yl]-2-propenal) | Colorectal cancer | NFATC3 | up | HCT116;HT29;SW480;LoVo | https://www.ncbi.nlm.nih.gov/pubmed/?term=24218372 |
| *Brassica* spp vegetables (cabbage, cauliflower, and brussels spouts) | Indole-3-carbinol (I3C) | Acute myeloid leukemia (AML) | NFKB1 | down | KBM-5 | https://www.ncbi.nlm.nih.gov/pubmed/?term=15811958 |
| *Naematoloma sublateritium*  (Hazel mushroom) | hexane fraction of N. sublateritium extract (HFNS) | Breast cancer | NFKB1 | down | MDA-MB‑231 | https://www.ncbi.nlm.nih.gov/pubmed/?term=24968898 |
| *Vaccinium macrocarpon* (Cranberry) | Polyphenolic compounds | Prostate cancer | NFKB1 | up | PC-3 | https://www.ncbi.nlm.nih.gov/pubmed/?term=17465224 |
| *Brassica* spp vegetables (cabbage, cauliflower, and brussels spouts) | Indole-3-carbinol (I3C) | Leukemia | NFKB1 | down | T-cell leukemia | https://www.ncbi.nlm.nih.gov/pubmed/?term=15811958 |
| *Camellia sinensis*  (Green tea) | Quercetin | Leukemia | BAX | up | HL-60 | https://www.ncbi.nlm.nih.gov/pubmed/?term=29472583 |
| *Hippophae rhamnoides* (Sea buckthorn) | Polyphenolic compounds | Prostate cancer | NFKB1 | up | PC-3 | https://www.ncbi.nlm.nih.gov/pubmed/?term=17465224 |
| *Ribes nigrum*  (Black currant) | Polyphenolic compounds | Prostate cancer | NFKB1 | up | PC-3 | https://www.ncbi.nlm.nih.gov/pubmed/?term=17465224 |
| *Teucrium alopecurus* | D-limonene; B-Phellandrene; A-Cadinol; T-Muurolol; A-Bisabolol | Leukemia | NFKB1 | down | KBM-5 | https://www.ncbi.nlm.nih.gov/pubmed/?term=28968948 |
| *Curcuma longa* (Turmeric) | Cucumin | Pancreatic cancer | NFKB1 | down | BxPC-3;Capan-1;Capan-2;ASPC-1;HS766-T | https://www.ncbi.nlm.nih.gov/pubmed/?term=15476283 |
| *Solanum lycopersicum* (Tomato) | A-tomatine | Prostate cancer | NFKB1 | down | PC-3 | https://www.ncbi.nlm.nih.gov/pubmed/?term=21541327 |
| *Ribes sativum*  (White currant) | Polyphenolic compounds | Prostate cancer | NFKB1 | up | PC-3 | https://www.ncbi.nlm.nih.gov/pubmed/?term=17465224 |
| *Rubus idaeus* (Raspberry) | Polyphenolic compounds | Prostate cancer | NFKB1 | up | PC-3 | https://www.ncbi.nlm.nih.gov/pubmed/?term=17465224 |
| *Cinnamomum verum* (Cinnamon) | 2-methoxycinnamaldehyde (2-MCA) | Lung cancer | NFKB1 | down | A549 | https://www.ncbi.nlm.nih.gov/pubmed/?term=26676220 |
| *Vaccinium myrtilloides* (Velvet leaf blueberry) | Polyphenolic compounds | Prostate cancer | NFKB1 | up | PC-3 | https://www.ncbi.nlm.nih.gov/pubmed/?term=17465224 |
| *Eriobotrya japonica* (Korean mandolin) | Εthyl ethanoate (EtOAc) | Lung cancer | NFKB1 | down | B16F19 | https://www.ncbi.nlm.nih.gov/pubmed/?term=21547674 |
| *Musa paradisiaca* (Banana) | Methanol extract of Musa paradisiaca inflorescence (PIMET) | Colon cancer | BAX | up | HT29 | https://www.ncbi.nlm.nih.gov/pubmed/?term=29243757 |
| *Curcuma longa* (Turmeric) | Cucumin | Bladder cancer | NFKB1 | down | KU-7 | https://www.ncbi.nlm.nih.gov/pubmed/?term=17363495 |
| *Amelanchier sanguinea* (Serviceberry) | Polyphenolic compounds | Prostate cancer | NFKB1 | up | PC-3 | https://www.ncbi.nlm.nih.gov/pubmed/?term=17465224 |
| *Laurus nobilis* (Bay laurel) | Lauroside B (megastigmane glycoside) | Melanoma | NFKB1 | down | A375;WM115;SK-Mel-28 | https://www.ncbi.nlm.nih.gov/pubmed/?term=21188975 |
| *Malus pumila* (Apple) | Phenolics; Quercetin | Breast cancer | NFKB1 | down | MCF-7 | https://www.ncbi.nlm.nih.gov/pubmed/?term=17373813 |
| *Ipomoea batatas*  (Sweet potato) | Sporamin | Pancreatic cancer | NFKB1 | down | PANC-1;BxPC-3 | https://www.ncbi.nlm.nih.gov/pubmed/?term=28714369 |
| *Brassica* spp vegetables (cabbage, cauliflower, and brussels spouts) | Indole-3-carbinol (I3C) | Acute myeloid leukemia (AML) | NOS2 | down | KBM-5 | https://www.ncbi.nlm.nih.gov/pubmed/?term=15811958 |
| *Brassica* spp vegetables (cabbage, cauliflower, and brussels spouts) | Indole-3-carbinol (I3C) | Leukemia | NOS2 | down | T-cell leukemia | https://www.ncbi.nlm.nih.gov/pubmed/?term=15811958 |
| *Juglans mandshruica* (Manchurian walnut) | Juglone | Leukemia | PARG | down | HL-60 | https://www.ncbi.nlm.nih.gov/pubmed/?term=22266044 |
| *Anguilla japonica* (Eel) | Eel skin mucus (ESM) | Leukemia | PARP1 | up | K562 | https://www.ncbi.nlm.nih.gov/pubmed/?term=26090845 |
| *Anacardium occidentale* (Cashews) | Cardanol monoene (CM) extracted from cashew nut shell liquid (CNSL) | Melanoma | PARP1 | up | M14 | https://www.ncbi.nlm.nih.gov/pubmed/?term=28627168 |
| *Holothuria edulis*  (Sea cucumber) | ESC-AQ (An aqueous fraction of the edible sea cucumber) | Leukemia | BAX | up | HL-60 | https://www.ncbi.nlm.nih.gov/pubmed/?term=23561113 |
| *Pulsatilla koreana* | Pulsatilla koreana extract (PKE) | Thyroid cancer | PARP1 | up | 8505c | https://www.ncbi.nlm.nih.gov/pubmed/?term=23135100 |
| *Semecarpus anacardium* (Marking nut) | Semecarpus anacardium nut extract (SA) | Breast cancer | PARP1 | up | T47D | https://www.ncbi.nlm.nih.gov/pubmed/?term=17572113 |
| *Citrus aurantium* | Flavonioids | Gastric cancer | PARP1 | up | RPMI-1640 | https://www.ncbi.nlm.nih.gov/pubmed/?term=22194772 |
| *Castanea* (Chestnut) | Ethanol extracts of raw chestnut (RCE) | Gastric cancer | PARP1 | up | AGS | https://www.ncbi.nlm.nih.gov/pubmed/?term=21779520 |
| Black Rice | Anthocyanin-rich extract of black rice (AEBR) | Breast cancer | PARP1 | up | MDA-MB-453 | https://www.ncbi.nlm.nih.gov/pubmed/?term=21058201 |
| *Rosmarinus ofﬁcinalis* (Rosemary) | Carnosic acid (CA) | Colon cancer | PARP1 | up | SW480;HT-29;HCT116 | https://www.ncbi.nlm.nih.gov/pubmed/?term=26152521 |
| *Panax quinquefolius* (American ginseng ) | Ginsenoside Rh2 (GRh2) | Colon cancer | PBK | down | HCT116 | https://www.ncbi.nlm.nih.gov/pubmed/?term=27746693 |
| *Musa paradisiaca* (Banana) | Methanol extract of Musa paradisiaca inflorescence (PIMET) | Colon cancer | PDHB | up | HT29 | https://www.ncbi.nlm.nih.gov/pubmed/?term=29243757 |
| Sanguinaria canadensis (Bloodroot) | Sanguinarine | Oral cancer | PIK3CD | down | KB | https://www.ncbi.nlm.nih.gov/pubmed/?term=27363951 |
| *Actinidia callosa* var. callosa | Ethyl acetate fraction of Actinidia callosa var. callosa (EAAC) | Hepatoma | PIK3CD | down | SK-Hep1 | https://www.ncbi.nlm.nih.gov/pubmed/?term=29356905 |
| *Asiasari radix* | Ethanol extract of Asiasari radix (EEAR) | Colon cancer | BAX | up | HCT-116 | https://www.ncbi.nlm.nih.gov/pubmed/?term=23255939 |
| *Juglans mandshruica* (Manchurian walnut) | Juglone | Leukemia | PIK3CD | down | HL-60 | https://www.ncbi.nlm.nih.gov/pubmed/?term=22266044 |
| *Cucurbita moschata* (Pumpkin) | Cucurmosin | Pancreatic cancer | PIK3CD | down | BxPC-3 | https://www.ncbi.nlm.nih.gov/pubmed/?term=22139427 |
| *Musa paradisiaca* (Banana) | Methanol extract of Musa paradisiaca inflorescence (PIMET) | Colon cancer | POLR2A | down | HT29 | https://www.ncbi.nlm.nih.gov/pubmed/?term=29243757 |
| *Musa paradisiaca* (Banana) | Methanol extract of Musa paradisiaca inflorescence (PIMET) | Colon cancer | POLR2C | down | HT29 | https://www.ncbi.nlm.nih.gov/pubmed/?term=29243757 |
| *Momordica charantia* (Bitter gourd ) | Fatty acids prepared from Bitter gourd oil (BGO-FFA) | Colon cancer | PPARG | up | CACO-2 | https://www.ncbi.nlm.nih.gov/pubmed/?term=15961301 |
| *Musa paradisiaca* (Banana) | Methanol extract of Musa paradisiaca inflorescence (PIMET) | Colon cancer | PRDX6 | down | HT29 | https://www.ncbi.nlm.nih.gov/pubmed/?term=29243757 |
| *Vaccinium macrocarpon* (Cranberry) | Polyphenolic compounds | Prostate cancer | PTGS2 | down | PC-3 | https://www.ncbi.nlm.nih.gov/pubmed/?term=17465224 |
| *Hippophae rhamnoides* (Sea buckthorn) | Polyphenolic compounds | Prostate cancer | PTGS2 | down | PC-3 | https://www.ncbi.nlm.nih.gov/pubmed/?term=17465224 |
| *Ribes sativum*  (White currant) | Polyphenolic compounds | Prostate cancer | PTGS2 | down | PC-3 | https://www.ncbi.nlm.nih.gov/pubmed/?term=17465224 |
| *Ribes nigrum*  (Black currant) | Polyphenolic compounds | Prostate cancer | PTGS2 | down | PC-3 | https://www.ncbi.nlm.nih.gov/pubmed/?term=17465224 |
| *Anacardium occidentale* (Cashews) | Cardanol monoene (CM) extracted from cashew nut shell liquid (CNSL) | Melanoma | BAX | up | M14 | https://www.ncbi.nlm.nih.gov/pubmed/?term=28627168 |
| *Rubus idaeus* (Raspberry) | Polyphenolic compounds | Prostate cancer | PTGS2 | down | PC-3 | https://www.ncbi.nlm.nih.gov/pubmed/?term=17465224 |
| *Aegle marmelos* | Βeta caryophyllene and caryophyllene oxide fractions of Aegle marmelos extract | Lymphoma | PTGS2 | down | IMR-32 | https://www.ncbi.nlm.nih.gov/pubmed/?term=24484210 |
| *Ribes hirtellum* (Gooseberry) | Polyphenolic compounds | Prostate cancer | PTGS2 | down | PC-3 | https://www.ncbi.nlm.nih.gov/pubmed/?term=17465224 |
| *Vaccinium myrtilloides* (Velvet leaf blueberry) | Polyphenolic compounds | Prostate cancer | PTGS2 | down | PC-3 | https://www.ncbi.nlm.nih.gov/pubmed/?term=17465224 |
| *Amelanchier sanguinea* (Serviceberry) | Polyphenolic compounds | Prostate cancer | PTGS2 | down | PC-3 | https://www.ncbi.nlm.nih.gov/pubmed/?term=17465224 |
| *Curcuma longa* (Turmeric) | Cucumin | Pancreatic cancer | PTGS2 | down | BxPC-3;Capan-1;Capan-2;ASPC-1;HS766-T | https://www.ncbi.nlm.nih.gov/pubmed/?term=15476283 |
| *Aegle marmelos* | Βeta caryophyllene and caryophyllene oxide fractions of Aegle marmelos extract | Neuroblastoma | PTGS2 | down | IMR-32 | https://www.ncbi.nlm.nih.gov/pubmed/?term=24484210 |
| *Curcuma longa* (Turmeric) | Cucumin | Bladder cancer | PTGS2 | down | KU-7 | https://www.ncbi.nlm.nih.gov/pubmed/?term=17363495 |
| *Brassica* spp vegetables (cabbage, cauliflower, and brussels spouts) | Indole-3-carbinol (I3C) | Acute myeloid leukemia (AML) | PTGS2 | down | KBM-5 | https://www.ncbi.nlm.nih.gov/pubmed/?term=15811958 |
| *Brassica* spp vegetables (cabbage, cauliflower, and brussels spouts) | Indole-3-carbinol (I3C) | Leukemia | PTGS2 | down | T-cell leukemia | https://www.ncbi.nlm.nih.gov/pubmed/?term=15811958 |
| Shallot | Flavonoid Isoliquiritigenin (ISL) | Cervical cancer | BAX | up | HeLa | https://www.ncbi.nlm.nih.gov/pubmed/?term=19536869 |
| *Basella rubra* (Spinach) | Natural antioxidants (NAOs) from spinach extract | Prostate cancer | RB1 | down | PC3 | https://www.ncbi.nlm.nih.gov/pubmed/?term=15327971 |
| *Basella rubra* (Spinach) | Natural antioxidants (NAOs) from spinach extract | Prostate cancer | RBL1 | down | PC3 | https://www.ncbi.nlm.nih.gov/pubmed/?term=15327971 |
| *Juglans mandshruica* (Manchurian walnut) | Juglone | Leukemia | ROS1 | up | HL-60 | https://www.ncbi.nlm.nih.gov/pubmed/?term=22266044 |
| *Persea declinata* (Bl.) | Persea declinata (Bl.) Kosterm bark methanolic crude extract (PDM) | Breast cancer | ROS1 | up | MCF-7 | https://www.ncbi.nlm.nih.gov/pubmed/?term=24808916 |
| *Foeniculum vulgare* (Fennel) | Chloroform fraction of fennel (CFF) | Breast cancer | ROS1 | up | MCF-7 | https://www.ncbi.nlm.nih.gov/pubmed/?term=29474902 |
| Red pepper | Capsaicin (trans-8-methyl-N-vanillyl-6-nonenamide) | Leukemia | ROS1 | up | HL-60 | https://www.ncbi.nlm.nih.gov/pubmed/?term=16827131 |
| *Anacardium occidentale* (Cashews) | Cardanol monoene (CM) extracted from cashew nut shell liquid (CNSL) | Melanoma | ROS1 | up | M14 | https://www.ncbi.nlm.nih.gov/pubmed/?term=28627168 |
| *Annona muricata* | Ethyl acetate extract of Annona muricata leaves (EEAM) | Colon cancer | ROS1 | up | HT-29;HTC-116 | https://www.ncbi.nlm.nih.gov/pubmed/?term=25195082 |
| *Vitis vinifera* (Grape) | Proanthocyanidin | Skin cancer | ROS1 | up | SCC12 | https://www.ncbi.nlm.nih.gov/pubmed/?term=28781636 |
| *Naematoloma sublateritium*  (Hazel mushroom) | hexane fraction of N. sublateritium extract (HFNS) | Breast cancer | SERPINE1 | up | MDA-MB‑231 | https://www.ncbi.nlm.nih.gov/pubmed/?term=24968898 |
| *Brassica* spp vegetables (cabbage, cauliflower, and brussels spouts) | Indole-3-carbinol (I3C) | Leukemia | AKT1 | down | T-cell leukemia | https://www.ncbi.nlm.nih.gov/pubmed/?term=15811958 |
| *Strychnos nux-vomica* (Loganiaceae) | Water extract of Strychni Semen (ESS) | Gastric cancer | BAX | up | RPMI-1640 | https://www.ncbi.nlm.nih.gov/pubmed/?term=18446845 |
| Red *Panax ginseng*  (Red Korean ginseng) | Korean red ginseng extract (KRGE) | Leukemia | SKP2 | down | NB4 | https://www.ncbi.nlm.nih.gov/pubmed/?term=24095829 |
| *Curcuma longa* (Turmeric) | Cucumin | Thyroid cancer | SMAD2 | down | BCPAP | https://www.ncbi.nlm.nih.gov/pubmed/?term=26826337 |
| Curcuma longa (Turmeric) | Cucumin | Thyroid cancer | SMAD3 | down | BCPAP | https://www.ncbi.nlm.nih.gov/pubmed/?term=26826337 |
| Chinese red yeast rice | Lovastatin (LV) | Colon cancer | SREBF2 | up | HCT-116;HT-29 | https://www.ncbi.nlm.nih.gov/pubmed/?term=17869085 |
| *Rosmarinus ofﬁcinalis* (Rosemary) | Carnosic acid (CA) | Colon cancer | STAT3 | down | SW480;HT-29;HCT116 | https://www.ncbi.nlm.nih.gov/pubmed/?term=26152521 |
| *Aloe vera* (Barbados aloe) | Aloin (AL) | Colorectal cancer | STAT3 | down | Fet;Geo;HCT116 | https://www.ncbi.nlm.nih.gov/pubmed/?term=23848964 |
| Kefir/Kefir grains | Lactobacillus brevis; Lactobacillus helveticus | Leukemia | TGFA | down | HTLV-1 | https://www.ncbi.nlm.nih.gov/pubmed/?term=21448298 |
| *Curcuma longa* (Turmeric) | Cucumin | Thyroid cancer | TGFB1 | down | BCPAP | https://www.ncbi.nlm.nih.gov/pubmed/?term=26826337 |
| Kefir/Kefir grains | Lactobacillus brevis; Lactobacillus helveticus | Leukemia | TGFB1 | up | HTLV-1 | https://www.ncbi.nlm.nih.gov/pubmed/?term=21448298 |
| *Naematoloma sublateritium*  (Hazel mushroom) | hexane fraction of N. sublateritium extract (HFNS) | Breast cancer | TIMP1 | up | MDA-MB‑231 | https://www.ncbi.nlm.nih.gov/pubmed/?term=24968898 |
| Celery | Celery seed extract (CSE) | Gastric cancer | BAX | up | BGC-823 | https://www.ncbi.nlm.nih.gov/pubmed/?term=22320960 |
| *Actinidia callosa* var. callosa | Ethyl acetate fraction of Actinidia callosa var. callosa (EAAC) | Hepatoma | TIMP1 | up | SK-Hep1 | https://www.ncbi.nlm.nih.gov/pubmed/?term=29356905 |
| *Actinidia callosa* var. callosa | Ethyl acetate fraction of Actinidia callosa var. callosa (EAAC) | Hepatoma | TIMP2 | up | SK-Hep1 | https://www.ncbi.nlm.nih.gov/pubmed/?term=29356905 |
| *Naematoloma sublateritium* (Hazel mushroom) | hexane fraction of N. sublateritium extract (HFNS) | Breast cancer | TIMP2 | up | MDA-MB‑231 | https://www.ncbi.nlm.nih.gov/pubmed/?term=24968898 |
| *Punica granatum* L. (Pomegranate) | Pomegranate peel extract (PoPx); Punicalagin; Ellagic acid | Prostate cancer | TIMP2 | up | DU145;PC3 | https://www.ncbi.nlm.nih.gov/pubmed/?term=28724216 |
| *Brassica* spp vegetables (cabbage, cauliflower, and brussels spouts) | Indole-3-carbinol (I3C) | Acute myeloid leukemia (AML) | TNF | down | KBM-5 | https://www.ncbi.nlm.nih.gov/pubmed/?term=15811958 |
| *Brassica* spp vegetables (cabbage, cauliflower, and brussels spouts) | Indole-3-carbinol (I3C) | Leukemia | TNF | down | T-cell leukemia | https://www.ncbi.nlm.nih.gov/pubmed/?term=15811958 |
| Powdered Milk | Lactobacillus spp | Colon cancer | TNF | down | ΗΤ-29;HCT116 | https://www.ncbi.nlm.nih.gov/pubmed/?term=25276792 |
| Bovine milk | Lactoferrin | Lung cancer | TNF | down | A549 | https://www.ncbi.nlm.nih.gov/pubmed/?term=23462173 |
| *Teucrium alopecurus* | D-limonene; B-Phellandrene; A-Cadinol; T-Muurolol; A-Bisabolol | Leukemia | TNF | down | KBM-5 | https://www.ncbi.nlm.nih.gov/pubmed/?term=28968948 |
| *Malus pumila* (Apple) | Phenolics; Quercetin | Breast cancer | TNF | down | MCF-7 | https://www.ncbi.nlm.nih.gov/pubmed/?term=17373813 |
| Pistacia vera L. (Pistachio) | Pistacia vera L. hulls extract (PVLH) | Breast cancer | BAX | up | MCF-7 | https://www.ncbi.nlm.nih.gov/pubmed/?term=29303970 |
| *Castanea* (Chestnut) | Ethanol extracts of raw chestnut (RCE) | Gastric cancer | TNFSF10 | up | AGS | https://www.ncbi.nlm.nih.gov/pubmed/?term=21779520 |
| *Prunus armeniaca* (Armenicae semen) | Amygdalin | Colon cancer | TOP1 | down | SNU-C4 | https://www.ncbi.nlm.nih.gov/pubmed/?term=16127745 |
| *Momordica charantia* (Bitter gourd ) | Fatty acids prepared from Bitter gourd oil (BGO-FFA) | Colon cancer | TP53 | up | CACO-2 | https://www.ncbi.nlm.nih.gov/pubmed/?term=15961301 |
| *Ocimum basilicum* (Sweet basil) | Lupeol epoxide | Breast cancer | TP53 | up | MCF-7;MDA-MB-231 | https://www.ncbi.nlm.nih.gov/pubmed/?term=25548920 |
| *Strychnos nux-vomica* (Loganiaceae) | Water extract of Strychni Semen (ESS) | Gastric cancer | TP53 | up | RPMI-1640 | https://www.ncbi.nlm.nih.gov/pubmed/?term=18446845 |
| *Arachis hypogaea* (Peanuts) | Resveratrol | Breast cancer | TP53 | up | MCF-7 | https://www.ncbi.nlm.nih.gov/pubmed/?term=27588384 |
| Red sorghum bran | 3-deoxyanthocyanin | Breast cancer | TP53 | up | MCF-7 | https://www.ncbi.nlm.nih.gov/pubmed/?term=23333825 |
| *Arachis hypogaea* (Peanuts) | Resveratrol | Cervical cancer | TP53 | up | HeLa | https://www.ncbi.nlm.nih.gov/pubmed/?term=27588384 |
| *Arachis hypogaea* (Peanuts) | Peanut skin procyanidins (PSP) | Prostate cancer | TP53 | up | DU145 | https://www.ncbi.nlm.nih.gov/pubmed/?term=29654773 |
| *Anacardium occidentale* (Cashews) | Cardanol monoene (CM) extracted from cashew nut shell liquid (CNSL) | Melanoma | TP53 | up | M14 | https://www.ncbi.nlm.nih.gov/pubmed/?term=28627168 |
| *Annona muricata* | Ethyl acetate extract of Annona muricata leaves (EEAM) | Colon cancer | BAX | up | HT-29;HTC-116 | https://www.ncbi.nlm.nih.gov/pubmed/?term=25195082 |
| *Allium hirtifolium* (Persian shallot) | 2-Methylpyridine-1-ium-1-sulfonate (MPS) | Breast cancer | TP53 | up | MCF-7;MDA-MB-231 | https://www.ncbi.nlm.nih.gov/pubmed/?term=28624423 |
| Shallot | Flavonoid Isoliquiritigenin (ISL) | Cervical cancer | TP53 | up | HeLa | https://www.ncbi.nlm.nih.gov/pubmed/?term=19536869 |
| *Asiasari radix* | Ethanol extract of Asiasari radix (EEAR) | Colon cancer | TP53 | up | HCT-116 | https://www.ncbi.nlm.nih.gov/pubmed/?term=23255939 |
| *Brassica* spp vegetables (cabbage, cauliflower, and brussels spouts) | Indole-3-carbinol (I3C) | Acute myeloid leukemia (AML) | TRAF1 | down | KBM-5 | https://www.ncbi.nlm.nih.gov/pubmed/?term=15811958 |
| *Brassica* spp vegetables (cabbage, cauliflower, and brussels spouts) | Indole-3-carbinol (I3C) | Leukemia | TRAF1 | down | T-cell leukemia | https://www.ncbi.nlm.nih.gov/pubmed/?term=15811958 |
| Bovine milk | Lactoferrin | Lung cancer | VEGFA | down | A549 | https://www.ncbi.nlm.nih.gov/pubmed/?term=23462173 |
| *Allium hirtifolium* (Persian shallot) | 2-Methylpyridine-1-ium-1-sulfonate (MPS) | Breast cancer | VEGFA | down | MCF-7;MDA-MB-231 | https://www.ncbi.nlm.nih.gov/pubmed/?term=28624423 |
| *Teucrium alopecurus* | D-limonene; B-Phellandrene; A-Cadinol; T-Muurolol; A-Bisabolol | Leukemia | VEGFA | down | KBM-5 | https://www.ncbi.nlm.nih.gov/pubmed/?term=28968948 |
| *Curcuma longa* (Turmeric) | Cucumin | Bladder cancer | VEGFA | down | KU-7 | https://www.ncbi.nlm.nih.gov/pubmed/?term=17363495 |
| *Aloe vera* (Barbados aloe) | Aloin (AL) | Colorectal cancer | VEGFA | down | Fet;Geo;HCT116 | https://www.ncbi.nlm.nih.gov/pubmed/?term=23848964 |
| *Arachis hypogaea* (Peanuts) | Peanut skin procyanidins (PSP) | Prostate cancer | BAX | up | DU145 | https://www.ncbi.nlm.nih.gov/pubmed/?term=29654773 |
| Black caraway | Thymoquinone (TQ) | Breast cancer | XIAP | down | MDA-MB-468;MDA-MB-231 | https://www.ncbi.nlm.nih.gov/pubmed/?term=24579801 |
| *Castanea* (Chestnut) | Ethanol extracts of raw chestnut (RCE) | Gastric cancer | XIAP | down | AGS | https://www.ncbi.nlm.nih.gov/pubmed/?term=21779520 |
| *Musa paradisiaca* (Banana) | Methanol extract of Musa paradisiaca inflorescence (PIMET) | Colon cancer | XIAP | down | HT29 | https://www.ncbi.nlm.nih.gov/pubmed/?term=29243757 |
| *Allium sativum* (Garlic) | N-benzyl-N-methyldecan-1-amine (NBNMA) | Leukemia | XIAP | down | U937 | https://www.ncbi.nlm.nih.gov/pubmed/?term=24859825 |
| *Laurus nobilis*  (Bay laurel) | Lauroside B (megastigmane glycoside) | Melanoma | XIAP | down | A375;WM115;SK-Mel-28 | https://www.ncbi.nlm.nih.gov/pubmed/?term=21188975 |
| *Brassica* spp vegetables (cabbage, cauliflower, and brussels spouts) | Indole-3-carbinol (I3C) | Leukemia | XIAP | down | T-cell leukemia | https://www.ncbi.nlm.nih.gov/pubmed/?term=15811958 |
| *Brassica* spp vegetables (cabbage, cauliflower, and brussels spouts) | Indole-3-carbinol (I3C) | Acute myeloid leukemia (AML) | XIAP | down | KBM-5 | https://www.ncbi.nlm.nih.gov/pubmed/?term=15811958 |
| *Portulaca oleracea* | Portulacerebroside A (PCA) | Leukemia | BAX | up | HL-60 | https://www.ncbi.nlm.nih.gov/pubmed/?term=26823708 |
| *Ipomoea batatas*  (Sweet potato) | Sporamin | Pancreatic cancer | BAX | up | PANC-1;BxPC-3 | https://www.ncbi.nlm.nih.gov/pubmed/?term=28714369 |
| *Polygonatum odoratum* (Vietnamese coriander) | Polygonatum odoratum extract | Breast cancer | BAX | up | MDA-MB-231 | https://www.ncbi.nlm.nih.gov/pubmed/?term=27698772 |
| *Punica granatum* L. (Pomegranate) | Pomegranate peel extract (PoPx); Punicalagin; Ellagic acid | Prostate cancer | BAX | up | DU145;PC3 | https://www.ncbi.nlm.nih.gov/pubmed/?term=28724216 |
| *Semecarpus anacardium* (Marking nut) | Semecarpus anacardium nut extract (SA) | Breast cancer | BAX | up | T47D | https://www.ncbi.nlm.nih.gov/pubmed/?term=17572113 |
| *Actinidia callosa* var. callosa | Ethyl acetate fraction of Actinidia callosa var. callosa (EAAC) | Hepatoma | AKT1 | down | SK-Hep1 | https://www.ncbi.nlm.nih.gov/pubmed/?term=29356905 |
| *Pulsatilla koreana* | Pulsatilla koreana extract (PKE) | Thyroid cancer | BAX | up | 8505c | https://www.ncbi.nlm.nih.gov/pubmed/?term=23135100 |
| *Allium sativum* (Garlic) | Allicin | Glioblastoma | BAX | up | U87MG | https://www.ncbi.nlm.nih.gov/pubmed/?term=22552443 |
| *Persea declinata* (Bl.) | Persea declinata (Bl.) Kosterm bark methanolic crude extract (PDM) | Breast cancer | BAX | up | MCF-7 | https://www.ncbi.nlm.nih.gov/pubmed/?term=24808916 |
| *Capparis spinosa* (Caperbush) | N-butanol extract of Capparis spinosa (CSBE) | Gastric cancer | BAX | up | SGC-7901 | https://www.ncbi.nlm.nih.gov/pubmed/?term=26668648 |
| *Crocus sativus* (Saffron) | Saffron extract (SE); Crocin | Prostate cancer | BAX | up | LAPC-4;PC3 | https://www.ncbi.nlm.nih.gov/pubmed/?term=23909737 |
| *Teucrium alopecurus* | D-limonene; B-Phellandrene; A-Cadinol; T-Muurolol; A-Bisabolol | Leukemia | BCL2 | down | KBM-5 | https://www.ncbi.nlm.nih.gov/pubmed/?term=28968948 |
| *Strychnos nux-vomica* (Loganiaceae) | Water extract of Strychni Semen (ESS) | Gastric cancer | BCL2 | down | RPMI-1640 | https://www.ncbi.nlm.nih.gov/pubmed/?term=18446845 |
| *Persea declinata* (Bl.) | Persea declinata (Bl.) Kosterm bark methanolic crude extract (PDM) | Breast cancer | BCL2 | down | MCF-7 | https://www.ncbi.nlm.nih.gov/pubmed/?term=24808916 |
| *Polygonatum odoratum* (Vietnamese coriander) | Polygonatum odoratum extract | Breast cancer | BCL2 | down | MDA-MB-231 | https://www.ncbi.nlm.nih.gov/pubmed/?term=27698772 |
| Red pepper | Capsaicin (trans-8-methyl-N-vanillyl-6-nonenamide) | Leukemia | BCL2 | down | HL-60 | https://www.ncbi.nlm.nih.gov/pubmed/?term=16827131 |
| *Daphne genkwa* | Yuanhuadine | Lung cancer | AKT1 | down | A549 | https://www.ncbi.nlm.nih.gov/pubmed/?term=21916433 |
| *Punica granatum* L. (Pomegranate) | Pomegranate peel extract (PoPx); Punicalagin; Ellagic acid | Prostate cancer | BCL2 | down | DU145;PC3 | https://www.ncbi.nlm.nih.gov/pubmed/?term=28724216 |
| *Allium hirtifolium* (Persian shallot) | 2-Methylpyridine-1-ium-1-sulfonate (MPS) | Breast cancer | BCL2 | down | MCF-7;MDA-MB-231 | https://www.ncbi.nlm.nih.gov/pubmed/?term=28624423 |
| Shallot | Flavonoid Isoliquiritigenin (ISL) | Cervical cancer | BCL2 | down | HeLa | https://www.ncbi.nlm.nih.gov/pubmed/?term=19536869 |
| *Crocus sativus* (Saffron) | Saffron extract (SE); Crocin | Prostate cancer | BCL2 | down | LAPC-4;PC3 | https://www.ncbi.nlm.nih.gov/pubmed/?term=23909737 |
| *Rosmarinus ofﬁcinalis* (Rosemary) | Carnosic acid (CA) | Colon cancer | BCL2 | down | SW480;HT-29;HCT116 | https://www.ncbi.nlm.nih.gov/pubmed/?term=26152521 |
| Black soybean | Flavanols; Phenolics acids; Anthocyanins; Isoflavones | Gastric cancer | BCL2 | down | AGS | https://www.ncbi.nlm.nih.gov/pubmed/?term=21462927 |
| Celery | Celery seed extract (CSE) | Gastric cancer | BCL2 | down | BGC-823 | https://www.ncbi.nlm.nih.gov/pubmed/?term=22320960 |
| *Vitex agnus-castus* (Vitex) | Ethanol extract of Vitex | Gastric cancer | BCL2 | down | KATO-III | https://www.ncbi.nlm.nih.gov/pubmed/?term=15833280 |
| *Cinnamomum verum* (Cinnamon) | 2-methoxycinnamaldehyde (2-MCA) | Lung cancer | BCL2 | down | A549 | https://www.ncbi.nlm.nih.gov/pubmed/?term=26676220 |
| *Momordica charantia* (Bitter gourd ) | Fatty acids prepared from Bitter gourd oil (BGO-FFA) | Colon cancer | BCL2 | down | CACO-2 | https://www.ncbi.nlm.nih.gov/pubmed/?term=15961301 |
| *Brassica* spp vegetables (cabbage, cauliflower, and brussels spouts) | Indole-3-carbinol (I3C) | Acute myeloid leukemia (AML) | AKT1 | down | KBM-5 | https://www.ncbi.nlm.nih.gov/pubmed/?term=15811958 |
| Red sorghum bran | 3-deoxyanthocyanin | Breast cancer | BCL2 | down | MCF-7 | https://www.ncbi.nlm.nih.gov/pubmed/?term=23333825 |
| *Angelica sinensis* | Acetone extract (AE-AS) | Lung cancer | BCL2 | down | A549 | https://www.ncbi.nlm.nih.gov/pubmed/?term=15261763 |
| *Allium sativum* (Garlic) | Allicin | Glioblastoma | BCL2 | down | U87MG | https://www.ncbi.nlm.nih.gov/pubmed/?term=22552443 |
| *Capparis spinosa* (Caperbush) | N-butanol extract of Capparis spinosa (CSBE) | Gastric cancer | BCL2 | down | SGC-7901 | https://www.ncbi.nlm.nih.gov/pubmed/?term=26668648 |
| *Pulsatilla koreana* | Pulsatilla koreana extract (PKE) | Thyroid cancer | BCL2 | down | 8505c | https://www.ncbi.nlm.nih.gov/pubmed/?term=23135100 |
| *Pistacia vera* L. (Pistachio) | Pistacia vera L. hulls extract (PVLH) | Breast cancer | BCL2 | down | MCF-7 | https://www.ncbi.nlm.nih.gov/pubmed/?term=29303970 |
| *Portulaca oleracea* | Portulacerebroside A (PCA) | Leukemia | BCL2 | down | HL-60 | https://www.ncbi.nlm.nih.gov/pubmed/?term=26823708 |
| *Semecarpus anacardium* (Marking nut) | Semecarpus anacardium nut extract (SA) | Breast cancer | BCL2 | down | T47D | https://www.ncbi.nlm.nih.gov/pubmed/?term=17572113 |
| *Anacardium occidentale* (Cashews) | Cardanol monoene (CM) extracted from cashew nut shell liquid (CNSL) | Melanoma | BCL2 | down | M14 | https://www.ncbi.nlm.nih.gov/pubmed/?term=28627168 |
| *Aegle marmelos* | Βeta caryophyllene and caryophyllene oxide fractions of Aegle marmelos extract | Lymphoma | BCL2 | down | IMR-32 | https://www.ncbi.nlm.nih.gov/pubmed/?term=24484210 |
| *Ananas comosus* (Pineapple) | Bromelain | Colorectal cancer | AKT1 | down | CACO-2;DLD-1 | https://www.ncbi.nlm.nih.gov/pubmed/?term=24123777 |
| *Annona muricata* | Ethyl acetate extract of Annona muricata leaves (EEAM) | Colon cancer | BCL2 | down | HT-29;HTC-116 | https://www.ncbi.nlm.nih.gov/pubmed/?term=25195082 |
| *Ocimum basilicum* (Sweet basil) | Lupeol epoxide | Breast cancer | BCL2 | down | MCF-7;MDA-MB-231 | https://www.ncbi.nlm.nih.gov/pubmed/?term=25548920 |
| *Ipomoea batatas*  (Sweet potato) | Sporamin | Pancreatic cancer | BCL2 | down | PANC-1;BxPC-3 | https://www.ncbi.nlm.nih.gov/pubmed/?term=28714369 |
| *Camellia sinensis*  (Green tea) | Quercetin | Leukemia | BCL2 | down | HL-60 | https://www.ncbi.nlm.nih.gov/pubmed/?term=29472583 |
| *Aegle marmelos* | Βeta caryophyllene and caryophyllene oxide fractions of Aegle marmelos extract | Neuroblastoma | BCL2 | down | IMR-32 | https://www.ncbi.nlm.nih.gov/pubmed/?term=24484210 |
| *Musa paradisiaca* (Banana) | Methanol extract of Musa paradisiaca inflorescence (PIMET) | Colon cancer | BCL2 | down | HT29 | https://www.ncbi.nlm.nih.gov/pubmed/?term=29243757 |
| *Arachis hypogaea* (Peanuts) | Peanut skin procyanidins (PSP) | Prostate cancer | BCL2 | down | DU145 | https://www.ncbi.nlm.nih.gov/pubmed/?term=29654773 |
| *Juglans mandshurica* (Walnut) | Juglanin | Breast cancer | BCL2 | down | MDA-MB-231;SKBR3;BT474;MCF-7 | https://www.ncbi.nlm.nih.gov/pubmed/?term=27899257 |
| *Asiasari radix* | Ethanol extract of Asiasari radix (EEAR) | Colon cancer | BCL2 | down | HCT-116 | https://www.ncbi.nlm.nih.gov/pubmed/?term=23255939 |
| *Allium sativum* (Garlic) | N-benzyl-N-methyldecan-1-amine (NBNMA) | Leukemia | BCL2 | down | U937 | https://www.ncbi.nlm.nih.gov/pubmed/?term=24859825 |
| *Black caraway* | Thymoquinone (TQ) | Breast cancer | AKT1 | down | MDA-MB-468;MDA-MB-231 | https://www.ncbi.nlm.nih.gov/pubmed/?term=24579801 |
| *Brassica* spp vegetables (cabbage, cauliflower, and brussels spouts) | Indole-3-carbinol (I3C) | Leukemia | BCL2 | down | T-cell leukemia | https://www.ncbi.nlm.nih.gov/pubmed/?term=15811958 |
| *Brassica* spp vegetables (cabbage, cauliflower, and brussels spouts) | Indole-3-carbinol (I3C) | Acute myeloid leukemia (AML) | BCL2 | down | KBM-5 | https://www.ncbi.nlm.nih.gov/pubmed/?term=15811958 |
| *Aloe vera* (Barbados aloe) | Aloin (AL) | Colorectal cancer | BCL2L1 | down | Fet;Geo;HCT116 | https://www.ncbi.nlm.nih.gov/pubmed/?term=23848964 |
| *Ipomoea batatas*  (Sweet potato) | Sporamin | Pancreatic cancer | BCL2L1 | down | PANC-1;BxPC-3 | https://www.ncbi.nlm.nih.gov/pubmed/?term=28714369 |
| *Camellia sinensis*  (Green tea) | Quercetin | Leukemia | BCL2L1 | down | HL-60 | https://www.ncbi.nlm.nih.gov/pubmed/?term=29472583 |
| *Holothuria edulis*  (Sea cucumber) | ESC-AQ (An aqueous fraction of the edible sea cucumber) | Leukemia | BCL2L1 | down | HL-60 | https://www.ncbi.nlm.nih.gov/pubmed/?term=23561113 |
| Shallot | Flavonoid Isoliquiritigenin (ISL) | Cervical cancer | BCL2L1 | down | HeLa | https://www.ncbi.nlm.nih.gov/pubmed/?term=19536869 |
| *Cinnamomum verum* (Cinnamon) | 2-methoxycinnamaldehyde (2-MCA) | Lung cancer | BCL2L1 | down | A549 | https://www.ncbi.nlm.nih.gov/pubmed/?term=26676220 |
| *Allium sativum* (Garlic) | N-benzyl-N-methyldecan-1-amine (NBNMA) | Leukemia | BCL2L1 | down | U937 | https://www.ncbi.nlm.nih.gov/pubmed/?term=24859825 |
| *Persea declinata* (Bl.) | Persea declinata (Bl.) Kosterm bark methanolic crude extract (PDM) | Breast cancer | BCL2L1 | down | MCF-7 | https://www.ncbi.nlm.nih.gov/pubmed/?term=24808916 |
